# Quantitative analysis of Chinese herbal medicine for post-stroke fatigue: a meta-analysis and medication pattern study

**DOI:** 10.3389/fphar.2026.1880065

**Published:** 2026-07-31

**Authors:** Yunlu Liu, Jiyue Wang, Xinyuan Wu, Yang Wang

**Affiliations:** 1 Sports Medicine Key Laboratory of Sichuan Province, Key Laboratory of Sports Medicine, General Administration of Sport of China, School of Sports Medicine and Health, Chengdu Sport University, Chengdu, China; 2 Postdoctoral Workstation, Affiliated Sport Hospital of Chengdu Sport University, Chengdu, China

**Keywords:** Chinese herbal medicine, fatigue, medication pattern, meta-analysis, stroke

## Abstract

**Background:**

Post-stroke fatigue (PSF) is a common and distressing condition among stroke survivors. Chinese herbal medicine (CHM) is frequently utilized in China to manage PSF. However, robust synthesized evidence supporting its efficacy remains limited. This study integrates meta-analysis with medication pattern analysis to establish a comprehensive evidence base for the rational clinical application of CHM in PSF treatment.

**Methods:**

Relevant RCTs were retrieved from eight databases-including PubMed, Cochrane Library, EMBASE, Web of Science, China National Knowledge Infrastructure Chinese Biomedical Database (SinoMed), Chinese Scientific Journal Database (VIP), and Wanfang-up to March 2026. Meta-analysis was performed using Review Manager 5.4, while funnel plot and Egger’s test were utilized to evaluate potential publication bias. Furthermore, we analyzed medication pattern in terms of usage frequency, properties, flavors, meridian tropism, and hierarchical cluster of Traditional Chinese Medicine actions of Chinese medicinal materials.

**Results:**

Nineteen trials involving 1,474 patients were included. Meta-analysis indicated that CHM significantly alleviated fatigue severity, as measured by fatigue severity scale. Evidence also suggested that CHM improved quality of life, motor function, and mood disorders. No significant publication bias was detected. Medication pattern analysis of 15 unique CHM formulas identified 52 Chinese medicinal materials, with high-frequency ingredients including Astragali radix, Angelicae sinensis radix, Chuanxiong rhizoma. The medicinal profiles were characterized by warm or slightly warm natures, sweet and pungent flavors, and tropism toward the spleen, liver, and heart meridians, reflecting core efficacies of tonifying deficiency and activating blood to resolve stasis.

**Conclusion:**

This study provides preliminary evidence for the clinical consideration of CHM in the management of post-stroke fatigue and highlights key Chinese medicinal materials and core TCM therapeutic strategies. However, further large-scale, multicenter randomized controlled trials are warranted to strengthen the evidence base and facilitate the integration of CHM into global clinical practice for post-stroke fatigue.

**Systematic Review Registration:**

https://www.crd.york.ac.uk/PROSPERO/view/CRD420261356354.

## 1 Introduction

Fatigue is a common and distressing symptom among stroke survivors, often persists even after other residual symptoms have disappeared (Cumming et al., 2015). Generally, post-stroke fatigue (PSF) manifests as a multidimensional experience characterized by a feeling of weariness, a lack of energy, and a reluctance to exert effort during physical and mental activities, and it cannot be relieved by rest (Lanctôt et al., 2020). With the global prevalence of PSF estimated as high as 47% (Zhan et al., 2023), the challenges it poses cannot be ignored.

Research on the pathophysiology of PSF is still in its infancy (English et al., 2024). As a complex subjective condition, PSF encompasses both physical and mental components; thus, it rarely occurs in isolation but is closely intertwined with motor dysfunction and emotional disturbances, such as anxiety and depression (Hubacher et al., 2012; Kutlubaev and Mead, 2011). PSF severely impairs quality of life and limits patients’ ability to perform daily activities (Li et al., 2026; Mandliya et al., 2016), which can lead to decreased motivation, social withdrawal, increased dependency, delayed rehabilitation progress, and an increased risk of mortality (Gyawali et al., 2022). Unfortunately, heightened mood deficits and reduced physical activity can in turn exacerbate PSF symptoms (Staub and Bogousslavsky, 2001; Lerdal et al., 2009; Wu et al., 2015a). Therefore, the implementation of effective PSF management strategies, focused either on fatigue symptoms *per se* or on the improvement of related comorbidities, is essential for the overall wellbeing of survivors (Wu et al., 2015b).

Regrettably, fatigue usually manifests gradually and is sometimes present during the later stages of recovery; it is frequently neglected as a “minor” symptom when co-existing with other severe sequelae (Williams et al., 1999). As a result, patients, caregivers and even the healthcare professionals tend not to address the symptom in a timely manner (Lanctôt et al., 2020). While advancements in acute stroke interventions have declined mortality rates, reducing fatigue severity remains a challenge. Strikingly, stroke survivors with excellent neurological recovery are often the most disabled by fatigue (Staub and Bogousslavsky, 2001; Wu et al., 2015b). Currently, pharmacological treatments like Modafinil, (−)-OSU6162 and antidepressants have been explored for relieving PSF; however, the evidence for their benefits remains insufficient or absent (Wu et al., 2015a; Choi-Kwon et al., 2007), and these treatments are associated with a higher rate of treatment-related side effects (Liang et al., 2025). Since the underlying causes of PSF are likely composed of biological, psychosocial and behavioral factors, non-pharmacological treatments-such as cognitive behavioral therapy, educational modalities, and physical training-may provide alternative approaches to alleviate fatigue in stroke patients. Nonetheless, these therapies are usually found to have beneficial effects on comorbid negative feelings or reduced physical fitness associated with PSF rather than the fatigue symptom itself (Zedlitz et al., 2012; Clarke et al., 2012). Moreover, the implementation of these approaches often necessitates professional supervision, specialized technical apparatus, and a sufficient baseline of neurological function, thereby restricting their acceptability and widespread accessibility (English et al., 2024).

Given these challenges, therapies incorporating Chinese herbal medicine (CHM) have attracted increasing attention due to their holistic therapeutic philosophy, ability to target multiple pathways and better cost-effectiveness (Liang et al., 2025; Li et al., 2025; Xu et al., 2020). Commonly used Chinese medicinal materials in CHM, including Astragali radix (Huang Qi) and Angelicae sinensis radix (Dang Gui), have been demonstrated to exert anti-fatigue functions and are often used to alleviate fatigue symptoms associated with exercise stress and various diseases, such as stroke (Choi et al., 2024; Yeh et al., 2014; Liu et al., 2016; Sheng et al., 2025). Regarding the Chinese herbal decoctions, the Buyang Huanwu decoction, is commonly used in Traditional Chinese Medicine (TCM) clinical settings to exert neuroprotective effect on stroke patients, and reduce fatigue severity among stroke survivors (Qin et al., 2025; Jin et al., 2021). In addition, a number of Chinese patent medicines (pharmaceutical products that are derived from CHM and approved by the National Medical Products Administration of China) have been used to treat PSF (He et al., 2017; Wu F. et al., 2019).

Despite the increasing integration of CHM into PSF treatment, robust scientific evidence to fully validate its efficacy remains insufficient, limiting its systematic development and utilization. Moreover, much remains unknown regarding the characteristics of CHM remedies in treating this condition. There is still a lack of universally recommended medications, and a better understanding of the spectrum of potential CHM strategies is warranted.

Therefore, this study aims to address these gaps by systematically analyzing the therapeutic effects, characteristics, and prescriptive patterns of CHM for PSF. By employing meta-analysis and medication pattern analysis, this study provides a quantitative assessment of the role of CHM in improving the clinical management of PSF patients, while also offering insights for the development of health-promoting products.

## 2 Materials and methods

For meta-analysis, this methodology adopted in this study adheres to the Preferred Reporting Items for Systematic Reviews and Meta-Analyses (PRISMA) guideline. The protocol was registered on PROSPERO with registered ID CRD420261356354 (available from https://www.crd.york.ac.uk/PROSPERO/view/CRD420261356354).

### 2.1 Search strategy

Both English and Chinese literature searches were performed in following the eight databases from their inception to March 2026: PubMed, Cochrane Library, EMBASE, Web of Science, China National Knowledge Infrastructure (CNKI), Chinese Biomedical Database (SinoMed), Chinese Scientific Journal Database (VIP), and Wanfang Database.

The search strategy employed a combined method of medical subject headings (MeSH) terms and free-text words (including “stroke”, “cerebrovascular disorders”, “fatigue”, “post-stroke fatigue”, “lassitude”, “traditional Chinese medicine”, “Chinese herbal medicine”, “Chinese patent medicine”, etc.) for comprehensive literature retrieval. Boolean operators “OR” and “AND” were used to combine search terms. Full search strategies for PubMed and CNKI are provided in Supplementary Table S1. Furthermore, we manually screened the reference lists of all included studies to identify any additional eligible publications.

### 2.2 Inclusion and exclusion criteria

#### 2.2.1 Types of studies

Eligibility for inclusion is restricted to English and Chinese language randomized controlled trials (RCTs), regardless of blinding or study setting.

#### 2.2.2 Types of participants

Patients diagnosed with stroke will be included, regardless of stroke type or demographic characteristics. Additionally, no restrictions were placed on the diagnostic criteria or the definition of PSF.

#### 2.2.3 Types of interventions

The intervention in the experimental group was CHM. The control groups included no treatment, placebo, or conventional treatment (CT). Additionally, studies comparing CHM plus CT versus CT alone were included.

In this review, CHM was defined as Chinese herbal prescriptions (containing one or more Chinese medicinal materials), herbal extracts, or Chinese patent medicines. There were no limitations on the dosage.

#### 2.2.4 Types of outcome measures

Primary outcomes: Fatigue was evaluated by various established tools, such as the Fatigue Severity Scale (FSS), Visual Analogue Scale for fatigue severity (VAS), and the Fatigue Assessment Instrument (FAI).

Secondary outcomes: Quality of life, such as the Barthel Index score (BI), Modified Barthel index (MBI), and Stroke Specific Quality of Life scale (SS-QOL); mood and motor functions were assessed by the Hamilton Anxiety Scale (HAMA), Hamilton Depression Rating Scale (HAMD), and Fugl-Meyer Assessment (FMA).

#### 2.2.5 Exclusion criteria

1. Non-RCTs.
2. Patients without a diagnosis of stroke.
3. Studies in which CHM was applied in both experimental and control groups. Studies that compared external treatment of Traditional Chinese Medicine (e.g., acupuncture, moxibustion, plaster or fumigation) or including additional active treatment strategies were also excluded.
4. Literature lacking essential data
5. Duplicate publications

### 2.3 Study selection and data extraction

For meta-analysis, following a predefined strategy, we initially gathered records from the aforementioned electronic databases and eliminated all duplicates. The screening process was conducted in two stages: an initial independent review of titles and abstracts by two researchers, followed by a comprehensive full-text evaluation of eligible candidates. A preliminary pilot phase involving three studies was utilized to refine a standardized extraction template. This tool facilitated the systematic gathering of information regarding study identifiers, participant demographics (such as gender distribution and age), intervention protocols (types of CHM, frequency and duration), and outcome results. To maintain data integrity, two authors performed the extraction independently, with a third investigator acting as an arbiter to resolve any discrepancies.

For studies with missing outcome data, attempts were made to obtain relevant data from the corresponding authors. If the missing data remained unavailable, no imputation was performed, and only the available data were included in the meta-analyses.

### 2.4 Methodological quality assessment

To determine the quality of the included literature, two authors performed a parallel assessment according to the Cochrane risk of bias 2 tool, which is an updated method for methodological assessment. The risk of bias was examined across five domains, encompassing randomization process, deviations from intended interventions, missing outcome data, measurement of the outcome, and selection of the reported result. Each domain was graded into three levels of risk: low, high, or some concerns. In instances of divergent opinions, the reviewers engaged in discussions to reach a unanimous agreement.

### 2.5 Statistical analysis

Meta-analysis was performed using Review Manager version 5.4. For the pooling of effects, mean differences (MD) and relative risks (RR) were employed for continuous and dichotomous variables, respectively, with 95% confidence intervals (CIs). Heterogeneity was quantified using the *I* ^*2*^ statistic. A fixed-effect model was utilized when *I* ^*2*^ < 50%, suggesting none of significant heterogeneity; otherwise, a random-effect model was favored when significant heterogeneity (I^2^ ≥ 50%) was observed. To investigate potential sources of inconsistency, subgroup analyses (based on the type of CHM formula) and sensitivity analyses using a leave-one-out (stepwise) method were conducted. Furthermore, publication bias was evaluated via funnel plots and Egger’s test for outcomes involving ten or more included trials.

### 2.6 Certainty of evidence assessment

The certainty of evidence for each outcome was assessed using the GRADEpro GDT (Grading of Recommendations Assessment, Development and Evaluation) web tool (https://www.gradepro.org/). The certainty of evidence was categorized into four levels: high, moderate, low, and very low.

### 2.7 Analysis of medication pattern

For medicine pattern analysis, an Excel database was established for the CHM formulas. Standardized names for each Chinese medicinal material were applied according to the Pharmacopoeia of the People’s Republic of China (2025 Edition). To facilitate data processing, each Chinese medicinal material was coded as a binary variable, where 1 indicated presence and 0 indicated absence within a formula. Data entry and verification were conducted independently by two investigators to minimize potential errors.

Medication pattern of CHM formulas was analyzed based on application frequency, properties, flavors, meridian tropism, and the hierarchical cluster of TCM actions of Chinese medicinal materials. SPSS 25.0 and Microsoft Excel 2022 were used for the statistical analysis of high-frequency ingredients (application frequency >20%). Additionally, radar charts and Sankey diagrams were employed for visual presentation.

## 3 Results

### 3.1 Study selection

**FIGURE 1:**
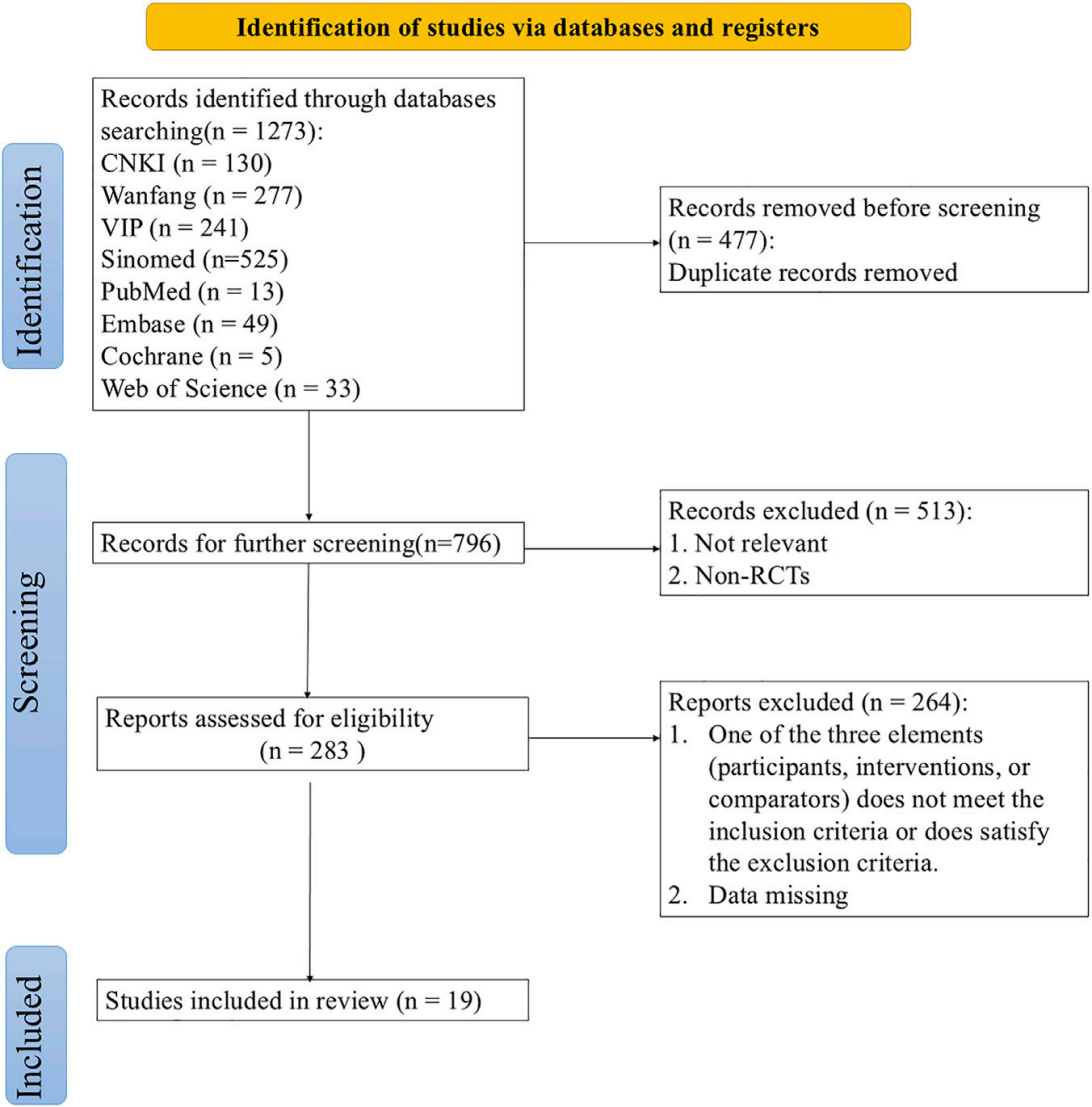
The flow chart of the study selection process.

As shown in the PRISMA flow diagram (Figure 1), the initial database search retrieved a total of 1,273 citations from the eight databases. After excluding 477 duplicate entries, 796 distinct records remained for further screening. During the title and abstract screening phase, 513 studies were excluded for not being RCTs or for lack of relevance. This led to a full-text appraisal of the remaining 283 manuscripts, of these, 264 records were subsequently excluded as they did not satisfy the pre-defined eligibility criteria. Ultimately, 19 RCTs were selected for inclusion in this meta-analysis (He et al., 2017; Wu F. et al., 2019; Li et al., 2013; Liang et al., 2016; Ye et al., 2018; Wen et al., 2013; Ye et al., 2016; Lin, 2020; Feng et al., 2014; Sima and Feng, 2017; Sun et al., 2014; Sun, 2018; Niu et al., 2013; Zhang and Song, 2024; Yang et al., 2020; Tan, 2014; Jiang et al., 2012; Liang et al., 2013; Pan et al., 2016).

### 3.2 Study characteristics

A total of 1,474 patients (733 in the experimental group and 741 in the control group) were enrolled across the 19 included studies, all of which were conducted in China. The participants were diagnosed with either hemorrhagic or ischemic stroke. The time since stroke onset varies across the included studies, ranging from the acute to the chronic phase, and the sample sizes of the individual trials ranged from 44 to 120. For interventions, the control groups received conventional treatment (CT), whereas the experimental groups were treated with CHM (including Chinese herbal decoctions or Chinese patent medicines) either alone or as an adjunct to CT.

Regarding outcome measures, the primary symptom of fatigue was assessed using the FSS in sixteen studies (He et al., 2017; Wu F. et al., 2019; Liang et al., 2016; Ye et al., 2018; Ye et al., 2016; Lin, 2020; Feng et al., 2014; Sima and Feng, 2017; Sun et al., 2014; Sun, 2018; Niu et al., 2013; Zhang and Song, 2024; Yang et al., 2020; Tan, 2014; Liang et al., 2013; Pan et al., 2016), the VAS in two studies (Wu F. et al., 2019; Pan et al., 2016), and the FAI in two studies (Li et al., 2013; Jiang et al., 2012). Quality of life among patients with PSF was evaluated using the BI in three studies (Tan, 2014; Jiang et al., 2012; Liang et al., 2013), the MBI in four studies (Feng et al., 2014; Sun et al., 2014; Yang et al., 2020; Pan et al., 2016), the SS-QOL in one study (Liang et al., 2016), and the energy subscale of the SS-QOL in five studies (Wu F. et al., 2019; Wen et al., 2013; Zhang and Song, 2024; Tan, 2014; Liang et al., 2013). Furthermore, to assess motor and mood functions, three studies reported the FMA (Wu F. et al., 2019; Liang et al., 2016; Feng et al., 2014), while two and three studies utilized the HAMA (Lin, 2020; Sima and Feng, 2017) and HAMD (Lin, 2020; Sima and Feng, 2017; Sun et al., 2014), respectively.

Among the 19 included studies, conventional therapy encompassed basic medical treatment, routine nursing care, rehabilitation training, and health education. Regarding the CHM interventions, three utilized Chinese patent medicines in their experimental groups (including Xiaopiling Granule, Erigeron Breviscapus Capsule, and Shengmai Injection). The remaining studies employed herbal decoctions, comprising practitioner-prescribed and classic Chinese prescriptions (e.g., Buyang Huanwu Decoction, Buzhong Yiqi Decoction, Xiao Xu Ming Decoction, and Dabu Gan Decoction, among others). The detailed profiles of the 19 eligible studies are presented in Table 1. And the detailed description of the composition of the CHM formulas are shown in Supplementary Table S2.

**TABLE 1:** Characteristics of included studies.

Study ID	Group	Number (M/F)	Age, years	Type of stroke	Time since stroke onset	Intervention and treatment duration	Form of CHM formula	Outcomes
Li et al. (2013)	Trial	32 (20/12)	65.8 ± 4.6	NR	≤ Half years	CT + Self-formulated herbal decoction, 1 dose/day, a total of 1 month	Chinese herbal decoction	FAI
	Control	34 (21/13)	65.2 ± 4.3			CT		
Liang et al. (2016)	Trial	46 (27/19)	66 ± 9.9	Hemorrhagic and ischemic	2.3 ± 0.9 months	CT + Buyang Huanwu decoction combined with modified Xiaoyao San, 1 dose/day, a total of 4 weeks	Chinese herbal decoction	FSS, FMA, SS-QOL
	Control	46 (26/20)	64.9 ± 11.6		2.5 ± 1.2 months	CT		
Ye et al. (2018)	Trial	33 (14/19)	63.85 ± 3.85	NR	NR	Buyang huanwu decoction, 1 dose/day, a total of 4 weeks	Chinese herbal decoction	FSS
	Control	30 (18/12)	62.79 ± 5.22			CT		
Wen et al. (2013)	Trial	33	NR	Hemorrhagic and ischemic	≤1 week	CT + modified Buyang huanwu decoction, 1 dose/day, a total of 2 weeks	Chinese herbal decoction	SS-QOL-E
	Control	32				CT		
Ye et al. (2016)	Trial	20 (11/9)	62.55 ± 5.85	NR	NR	Modified Buyang huanwu decoction, 1 dose/day, a total of 4 weeks	Chinese herbal decoction	FSS
	Control	24 (12/12)	64.79 ± 4.22			CT		
Lin (2020)	Trial	31 (19/12)	64.12 ± 6.71	NR	24.37 ± 8.13 days	CT + buzhong yiqi decoction, 1 dose/day, a total of 4 weeks	Chinese herbal decoction	FSS, HAMA, HAMD
	Control	31 (21/10)	63.42 ± 5.56		22.42 ± 7.33 days	CT		
Feng et al. (2014)	Trial	57 (35/22)	63.9 ± 9.5	Hemorrhagic and ischemic	17.7 ± 10.7 days	CT + buzhong yiqi decoction, 1 dose/day, a total of 4 weeks	Chinese herbal decoction	FSS, FMA, MBI
	Control	58 (35/23)	65.2 ± 8.8		16.4 ± 10.6 days	CT		
Sima and Feng (2017)	Trial	40 (23/17)	65.9 ± 7.57	Hemorrhagic and ischemic	23.4 ± 7.87 days	CT + buzhong yiqi decoction, 1 dose/day, a total of 4 weeks	Chinese herbal decoction	FSS, HAMA, HAMD
	Control	40 (25/15)	65.45 ± 8.22		23.65 ± 8.77 days	CT		
Sun et al. (2014)	Trial	38 (20/18)	55.6 ± 8.8	Hemorrhagic and ischemic	3 ± 0.3 months	CT + Dabu Gan decoction, 1 dose/day, a total of 4 weeks	Chinese herbal decoction	FSS, HAMD, MBI
	Control	45 (25/20)	54.8 ± 9.1		3 ± 0.5 months	CT		
Wu F. et al. (2019)	Trial	26 (14/12)	64 ± 5	NR	NR	CT + Erigeron Breviscapus Capsule, 3 capsules per time, three times daily, for a total of 60 days	Chinese patent medicine	FSS, FMA SS-QOL, VAS
	Control	26 (13/13)	65 ± 4			CT		
Sun (2018)	Trial	42	62.7 ± 4.2	Ischemic	2.5 ± 1 month	CT + modified Buyang huanwu decoction, 1 dose/day, a total of 4 weeks	Chinese herbal decoction	FSS
	Control	42	62.3 ± 4.1		2.6 ± 0.9 months	CT		
He et al. (2017)	Trial	24	58–81	Ischemic	NR	CT + Shengmai Injection, 1 dose/day, a total of 14 days	Chinese patent medicine	FSS
	Control	24				CT		
Niu et al. (2013)	Trial	30 (20/10)	49–72	NR	NR	CT + Xiaopiling Granule, 6 g per time, three times daily, for a total of 4 weeks	Chinese patent medicine	FSS
	Control	30 (22/8)	50–73			CT		
Zhang et al. (2024)	Trial	36 (33/3)	56.17 ± 11.90	Hemorrhagic and ischemic	70.00 ± 38.90 days	CT + Xiao Xu Ming decoction, 1 dose/day, a total of 4 weeks	Chinese herbal decoction	FSS, SS-QOL-E
	Control	36 (34/2)	56.61 ± 16.21		73.00 ± 38.02 days	CT		
Yang et al. (2020)	Trial	30 (21/9)	54 ± 9	Hemorrhagic and ischemic	1.26 ± 0.93 months	CT + yiqi Fuyang decoction, 1 dose/day, a total of 4 weeks	Chinese herbal decoction	FSS, MBI
	Control	30 (18/12)	55 ± 7		1.32 ± 0.81 months	CT		
Tan (2014)	Trial	48 (26/22)	65 ± 5.67	Hemorrhagic and ischemic	1.8 ± 0.2 years	CT + Yulang Xiaopi decoction, 1 dose/day, a total of 4 weeks	Chinese herbal decoction	FSS, SS-QOL-E, BI
	Control	48 (25/23)	64 ± 5.93		1.9 ± 0.4 years	CT		
Jiang et al. (2012)	Trial	60 (39/21)	65.9 ± 6.1	NR	NR	CT + Shuangbu decoction, 1 dose/day, a total of 4 weeks	Chinese herbal decoction	BI, FAI
	Control	60 (33/27)	68.4 ± 6.5			CT		
Liang et al. (2013)	Trial	60 (32/28)	68.7 ± 6.4	Hemorrhagic and ischemic	20.6 ± 2.8 months	CT + Yulang Xiaopi decoction, 1 dose/day, a total of 4 weeks	Chinese herbal decoction	FSS, SS-QOL-E, BI
	Control	60 (35/25)	67.7 ± 7.3		22.8 ± 3.6 months	CT		
Pan et al. (2016)	Trial	47 (26/21)	62.74 ± 8.98	Hemorrhagic and ischemic	73.23 ± 32.62 days	CT + Buyang huanwu decoction, 1 dose/day, a total of 4 weeks	Chinese herbal decoction	FSS, VAS, MBI
	Control	45 (24/21)	63.40 ± 9.31		72.18 ± 28.10 days	CT		

Abbreviations: FAI, fatigue assessment instrument; FSS, fatigue severity scale; VAS, visual analogue scale for fatigue severity; BI, barthel index; MBI, modified barthel index; SS-QOL, stroke specific quality of life scale; SS-QOL-E, Stroke Specific Quality of Life scale-energy domain; HAMA, hamilton anxiety scale; HAMD, hamilton depression rating scale; CT, conventional therapy; CHM, chinese herbal medicine; F, female; M, male; NR, not reported.

### 3.3 Risk of bias

**FIGURE 2:**
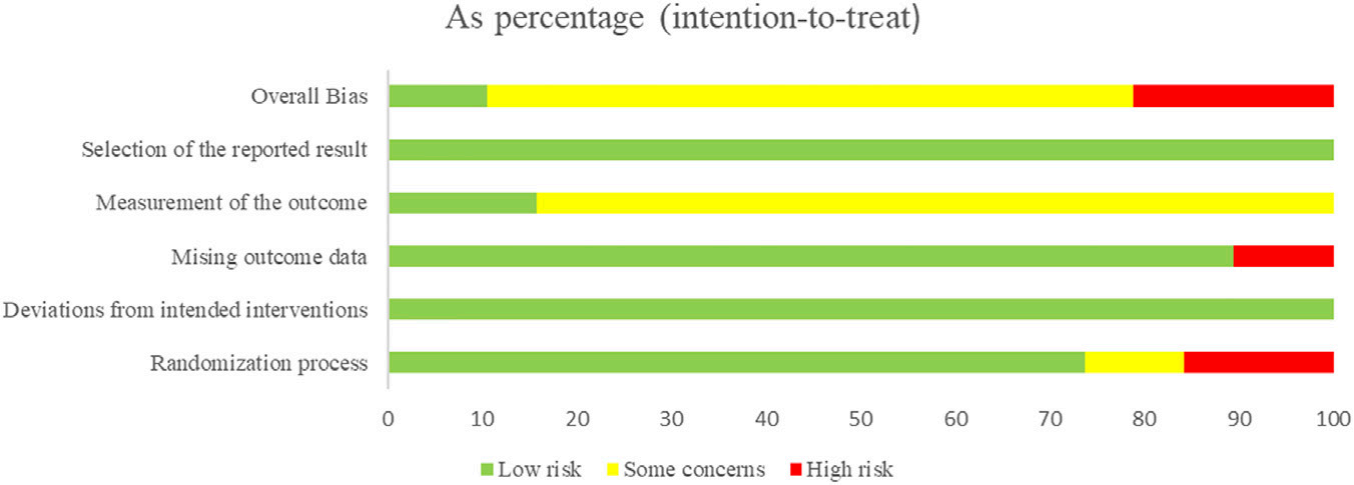
Overview of risk of bias assessment for included studies. Bars represent the percentage of studies rated as low risk (green), some concerns (yellow), or high risk (red).

Figure 2 provides an overview of the risk of bias assessment for the included studies, while Supplementary Figure S1 details the results for each individual study. Among the 19 included trials, 2 were rated as having a low overall risk of bias, 13 raised some concerns, and four were rated as high risk. The most frequent cause of moderate bias concerns was insufficient information regarding the blinding of outcome assessors, suggesting a potential for detection bias that could influence the findings. High risk of bias was typically due to improper randomization processes and data omissions. Overall, these issues in the reporting domains raise some concerns regarding the risk of bias.

### 3.4 Effect of CHM on fatigue

The severity of fatigue symptom was assessed by various tools, including FSS, VAS, and FAI.

#### 3.4.1 FSS

**FIGURE 3:**
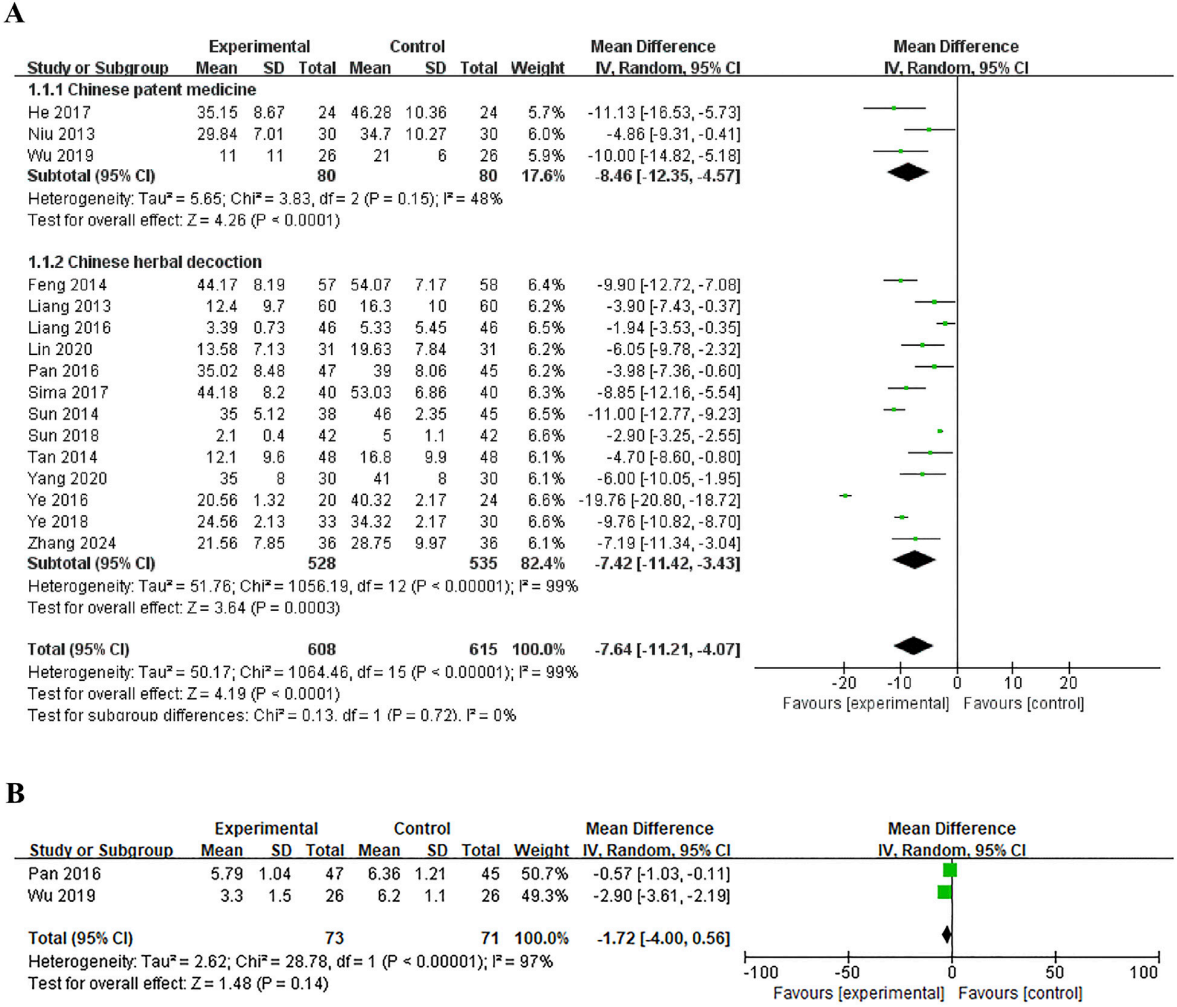
Forest plot of fatigue severity scores in patients receiving CHM versus the control group. **(A)** FSS; **(B)** VAS.

Sixteen studies assessed fatigue by using FSS (He et al., 2017; Wu F. et al., 2019; Liang et al., 2016; Ye et al., 2018; Ye et al., 2016; Lin, 2020; Feng et al., 2014; Sima and Feng, 2017; Sun et al., 2014; Sun, 2018; Niu et al., 2013; Zhang and Song, 2024; Yang et al., 2020; Tan, 2014; Liang et al., 2013; Pan et al., 2016). Among which, one (Lin, 2020) reported FSS scores using the same scale content but in the opposite direction (i.e., scores decreased as symptom severity increased). In accordance with the Cochrane Handbook for Systematic Reviews of Interventions (Higgins et al., 2024), we performed data adjustment by subtracting the mean score from the maximum theoretical value of the scale. The heterogeneity test revealed significant statistical variation among the included studies (*p* < 0.00001, *I* ^*2*^ = 99%); therefore, a random-effects model was utilized for meta-analysis. The pooled results demonstrated a significant lower FSS scores in the experimental group compared to the control group (MD = −7.64; 95% CI: 11.21, −4.07; *p* < 0.0001). Given the high heterogeneity, sixteen trials were sub-grouped into Chinese patent medicine (standardized, mass-produced pharmaceutical prescriptions) and Chinese herbal decoction (individualized decoctions derived from classic or practitioner-prescribed prescriptions). Among the three trials utilizing Chinese patent medicine, the subgroup analysis showed a significant positive effect (MD = −8.46; 95% CI: 12.35, −4.57; *p* < 0.0001) with no significant heterogeneity (*p* = 0.15, *I* ^*2*^ = 48%); for the remaining studies evaluating Chinese herbal decoction, fatigue severity was still significantly lower in the experimental group than that in the control (MD = −7.42; 95% CI: 11.42, −3.43; *p* = 0.0003), but substantial heterogeneity persisted (*p* < 0.00001, *I* ^*2*^ = 99%) (Figure 3A).

#### 3.4.2 VAS

Two studies reported the VAS (Wu F. et al., 2019; Pan et al., 2016). Statistical analysis revealed significant heterogeneity between these studies (*p* < 0.00001, *I* ^*2*^ = 97%). Using a random-effects model, the pooled results showed that the experimental groups did not achieve a significant improvement in VAS scores compared to the control group (MD = −1.72; 95% CI: 4.00, 0.56; *p* = 0.14) (Figure 3B).

#### 3.4.3 FAI

As the FAI outcomes reported in the included studies were unsuitable for quantitative meta-analysis, a narrative description was conducted. Two studies recorded the FAI. In Li’s study (Li et al., 2013), the result was reported as the proportions of individuals with varying levels of fatigue based on FAI score; the experimental group showed a lower proportion of participants with moderate-to-severe symptoms than the control group. Similarly, Jiang et al. (2012) recorded the specific number of participants in each score range, finding that patients receiving CHM treatment were predominantly concentrated in the lower score ranges, with fewer patients fell into the higher score categories relative to the control group.

### 3.5 Effect of CHM on quality of life

Quality of Life (QoL) assessment provides a comprehensive evaluation of patients’ daily lives, including physical function, psychological status, social roles, and subjective perceptions across the included groups. Therefore, it was selected as one of our outcome measures. The evaluation tools employed to capture these multidimensional aspects include the SS-QOL (or SS-QOL-E), BI, and MBI.

#### 3.5.1 SS-QOL and SS-QOL-E

One study reported changes in the total SS-QOL scores (Liang et al., 2016), which indicated a significant improvement in CHM-treated patients.

**FIGURE 4:**
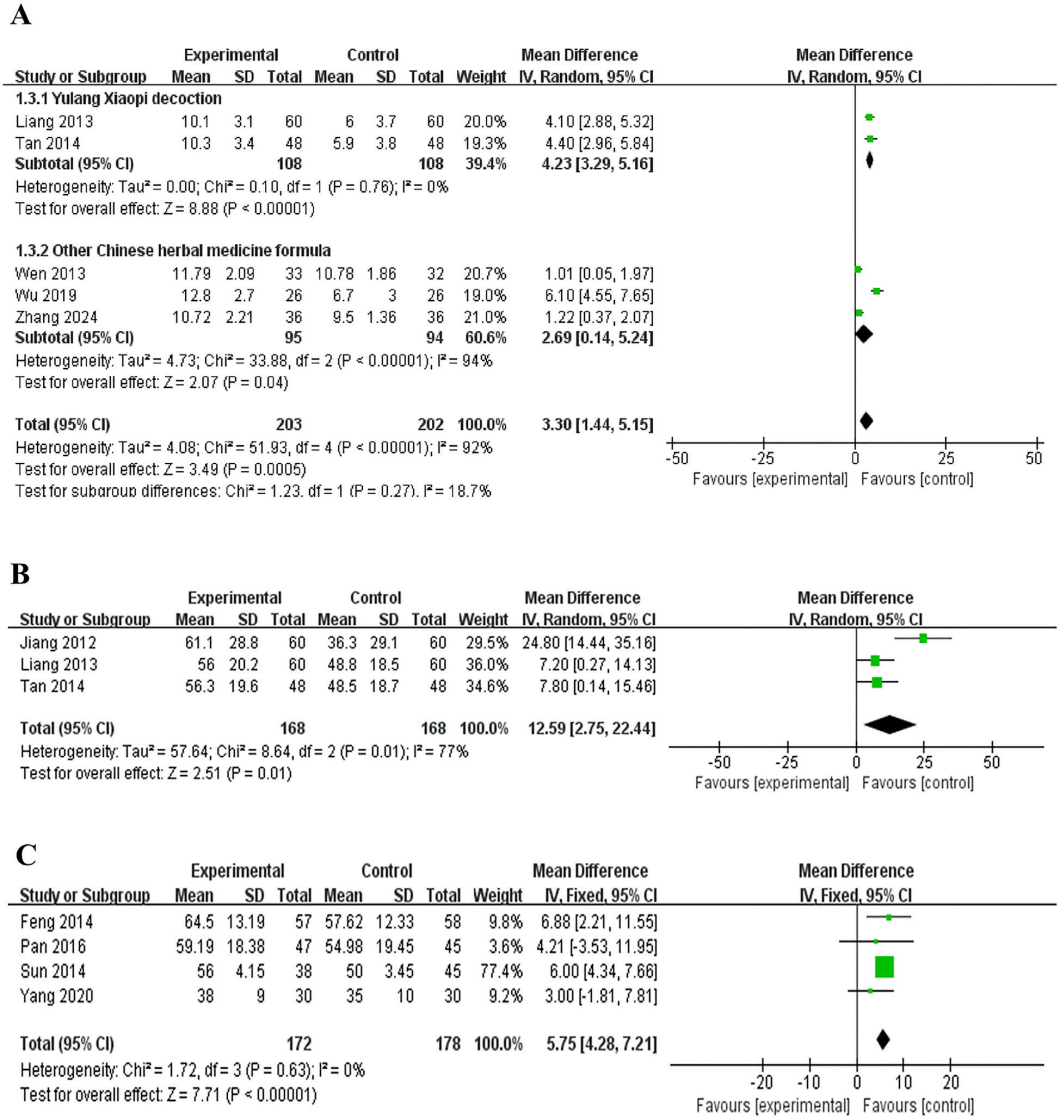
Forest plot of quality-of-life scores in patients receiving CHM versus the control group. **(A)** SS-QOL-E; **(B)** BI; **(C)** MBI.

Five studies employed the SS-QOL energy domain (SS-QOL-E) as an outcome indicator (Wu F. et al., 2019; Wen et al., 2013; Zhang and Song, 2024; Tan, 2014; Liang et al., 2013). Figure 4A illustrates the meta-analysis of the effect of CHM treatment versus control on SS-QOL-E scores. The CHM group achieved significantly higher SS-QOL-E scores compared to the control group (MD = 3.30; 95% CI: 1.44, 5.15; *p* = 0.0005), though significant heterogeneity was observed (*p* < 0.00001, *I* ^*2*^ = 92%). To explore this, a subgroup analysis was performed based on the type of CHM intervention: Yulang Xiaopi decoction versus other CHM formulas. For the two studies using Yulang Xiaopi decoction, the sub-pooled results showed a significant improvement in SS-QOL-E scores (MD = 4.23; 95% CI: 3.29, 5.16; *p* < 0.00001) with no statistical heterogeneity (*p* = 0.76, *I* ^*2*^ = 0%). In contrast, while the subgroup using other CHM formulas also showed a positive effect (MD = 2.69; 95% CI: 0.14, 5.24; *p* = 0.04), substantial statistical heterogeneity remained (*p* < 0.00001, *I* ^*2*^ = 94%).

#### 3.5.2 BI and MBI

A total of three studies reported the BI score (Tan, 2014; Jiang et al., 2012; Liang et al., 2013). Significant heterogeneity was observed among studies (*p* = 0.01, *I* ^*2*^ = 77%). Using a random-effects model, the results indicated that CHM intervention significantly improved BI scores (MD = 12.59; 95% CI: 2.75, 22.44; *p* = 0.01). As illustrated in Figure 4B. Sensitivity analysis showed that after excluding Jiang’ study (Jiang et al., 2012), the *I* ^*2*^ value dropped to 0%, while the overall effect remained consistent, indicating that Jiang’s study was the primary source of the high heterogeneity.

For MBI measurement, four studies were included into meta-analysis (Feng et al., 2014; Sun et al., 2014; Yang et al., 2020; Pan et al., 2016). The pooled results demonstrated that there was significant positive effect in favor of the CHM intervention group (MD = 5.75; 95% CI: 4.28, 7.21; *p* < 0.00001) with no significant heterogeneity (*p* = 0.63, *I* ^*2*^ = 0%). As shown in Figure 4C.

### 3.6 Effect of CHM on mood and motor functions

Patients with PSF often present with decreased motor function and mood disorders (such as anxiety and depression). Therefore, FMA, HAMA, and HAMD were included as outcome measures.

#### 3.6.1 FMA

**FIGURE 5:**
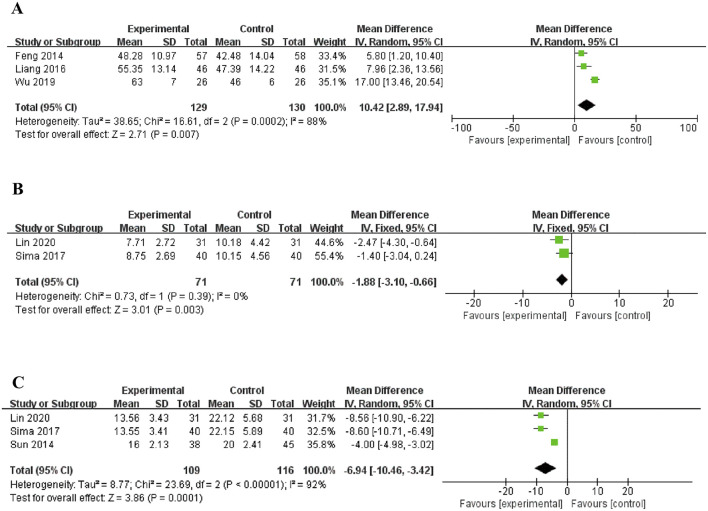
Forest plot of mood and motor function scores in patients receiving CHM versus the control group. **(A)** FMA; **(B)** HAMA; **(C)** HAMD.

A total of three studies mentioned FMA scores (Wu F. et al., 2019; Liang et al., 2016; Feng et al., 2014). As illustrated in Figure 5A, the CHM group achieved significantly higher FMA scores compared to the control group at the end of treatment (MD = 10.42; 95% CI: 2.89, 17.94; p = 0.007), although significant heterogeneity was present (*p* = 0.0002, *I* ^*2*^ = 88%). Sensitivity analysis revealed that the *I* ^*2*^ decreased to 0% after excluding Wu’s study (Wu F. et al., 2019), and the pooled overall effect remained stable, suggesting this trial was the main contributor to high heterogeneity.

#### 3.6.2 HAMA

Two studies applied the HAMA to assess the degree of anxiety after invention (Lin, 2020; Sima and Feng, 2017). Patients in the CHM group showed lower HAMA score than that in the control group (MD = −1.88; 95% CI: 3.10, −0.66; *p* = 0.003) with no statistical heterogeneity (*p* = 0. 39, *I* ^*2*^ = 0%) (Figure 5B).

#### 3.6.3 HAMD

Three studies utilized the HAMD to assess the degree of depression (Lin, 2020; Sima and Feng, 2017; Sun et al., 2014). The pooled results showed that HAMD scores were significantly lower in the experimental group compared to the control (MD = −6.94; 95% CI: 10.46, −3.42; *p* = 0.0001), although significant heterogeneity was observed (*p* < 0.0001, *I* ^*2*^ = 92%). As illustrated in Figure 5C. Upon the removal of Sun’s study (Sun et al., 2014) in sensitivity analysis, the *I* ^*2*^ fell to 0% with no obvious change in the overall effect, indicating that this study was the primary cause of heterogeneity. The mean time since stroke onset in Sun’s study was approximately 3 months, whereas that in the other two studies was around 20 days (Table 1). This difference may have contributed to this heterogeneity.

### 3.7 Publication bias

A funnel plot was generated to visualize publication bias across the 16 studies that utilized the FSS as an outcome measure, as illustrated in Supplementary Figure S2. Egger’s test indicated no statistically significant risk of publication bias (95%CI: 9.48, 2.15; *P* = 0.1983).

### 3.8 Certainty of evidence

The GRADE assessment indicated that the certainty of evidence ranged from moderate to very low across all assessed outcomes. The certainty of evidence for the VAS and HAMA outcomes was downgraded to very low and low, respectively, because of the risk of bias, unexplained heterogeneity, and small total sample size. The certainty of evidence for the remaining outcomes was rated as moderate because of concerns regarding the risk of bias (Supplementary Table S3).

### 3.9 Medication pattern of CHM

Furthermore, to explore in depth the underlying medication patterns of CHM formulas for treating PSF, relevant data on usage frequency, properties, flavors, meridian tropism, and the TCM effects of the Chinese medicinal materials were collected and analyzed.

By reviewing the CHM interventions across the 19 included studies and eliminating duplicate prescriptions, 15 CHM formulas with different compositions of Chinese medicinal materials were identified. Across the 15 CHM formulas (comprising Chinese patent medicines, practitioner-prescribed and classic Chinese prescriptions), a total of 52 distinct ingredients were identified following name standardization. Cumulatively, these Chinese medicinal materials occurred 135 times across all formulas. Notably, 11 Chinese medicinal materials demonstrated a usage frequency exceeding 20%, with Astragali radix, Angelicae sinensis radix, Chuanxiong rhizoma being the top three most frequently used ingredients. The frequency counts and corresponding usage proportions of these frequently prescribed Chinese medicinal materials are presented in detail in Table 2.

**TABLE 2:** Chinese medicinal material with frequency exceeding 20%.

Rank	Ingredients	Taxonomic validation	Frequency	Percentage
1	Astragali radix	*Astragalus membranaceus* (Fisch.) Bge. [Fabaceae; Astragali radix]	10	66.67%
2	Angelicae sinensis radix	*An*g*elica sinensis* (Oliv.) Diels [Apiaceae; Angelicae sinensis radix]	10	66.67%
3	Chuanxiong rhizoma	*Ligusticum chuanxiong* Hort. [Apiaceae; Chuanxiong rhizoma]	9	60%
4	Paeoniae radix rubra	*Paeonia lactiflora* Pall. [Paeoniaceae; Paeoniae radix rubra]	8	53.33%
5	Pheretima	*Pheretima aspergillum* (E. Perrier) [Megascolecidae; Pheretima]	7	46.67%
6	Persicae semen	*Prunus persica* (L.) Batsch [Rosaceae; Persicae semen]	6	40%
7	Carthami flos	*Carthamus tinctorius* L. [Asteraceae; Carthami flos]	6	40%
8	Atractylodis macrocephalae rhizoma	*Atractylodes macrocephala* Koidz. [Asteraceae; Atractylodis macrocephalae rhizoma]	5	33.33%
9	Cimicifugae rhizoma	*Cimicifuga foetida* L. [Ranunculaceae; Cimicifugae rhizoma]	4	26.67%
10	Glycyrrhizae radix et rhizoma	*Glycyrrhiza uralensis* Fisch. [Fabaceae; Glycyrrhizae radix et rhizoma]	4	26.67%
11	Ginseng radix et rhizoma	*Panax ginseng* C. A. Mey. [Araliaceae; Ginseng radix et rhizoma]	4	26.67%

**FIGURE 6:**
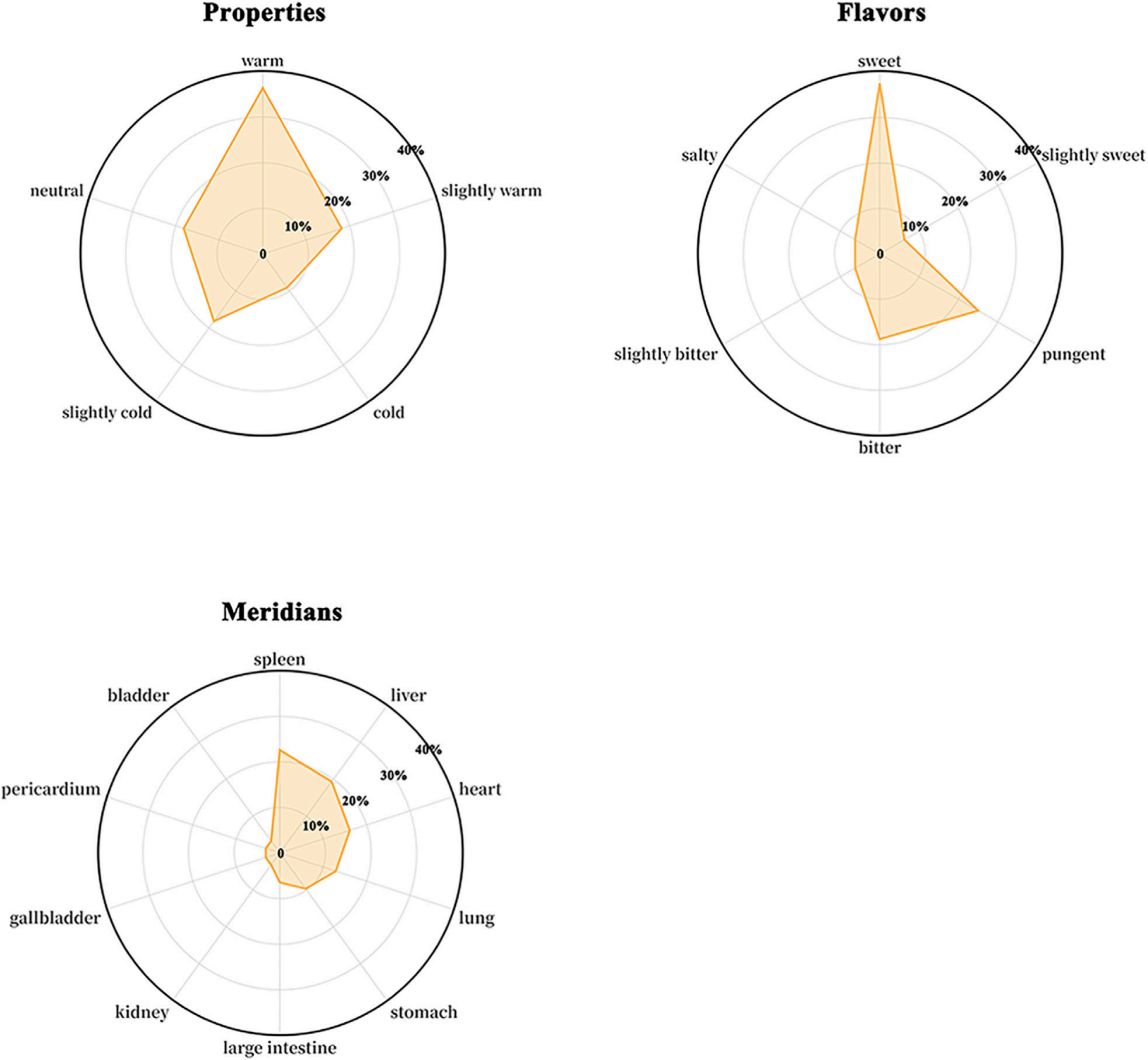
Properties, flavors, and meridian tropisms of high-frequency Chinese medicinal materials for post-stroke fatigue.

Regarding the properties, flavors and meridian tropism of Chinese medicinal materials with a usage frequency >20%, the analysis was conducted according to the *Chinese Pharmacopoeia* (Version 2025). Initially, five properties were identified: warm (36.36%), slightly warm (18.18%), neutral (18.18%), slightly cold (18.18%), and cold (9.09%). No cool or hot properties were observed among the analyzed ingredients. The Chinese medicinal materials exhibited six flavors, with sweet (37.5%) being the most prevalent, followed by pungent (25%), bitter (18.75%), slightly bitter (6.25%), slightly sweet (6.25%), salty (6.25%). The meridian tropism analysis covered ten organs. The spleen (22.58%), liver (19.35%), and heart (16.13%) were the most commonly targeted, followed by the lung (12.90%), stomach (9.68%), large intestine (6.45%), kidney (3.23%), gallbladder (3.23%), pericardium (3.23%), and bladder (3.23%), as shown in Figure 6.

**FIGURE 7:**
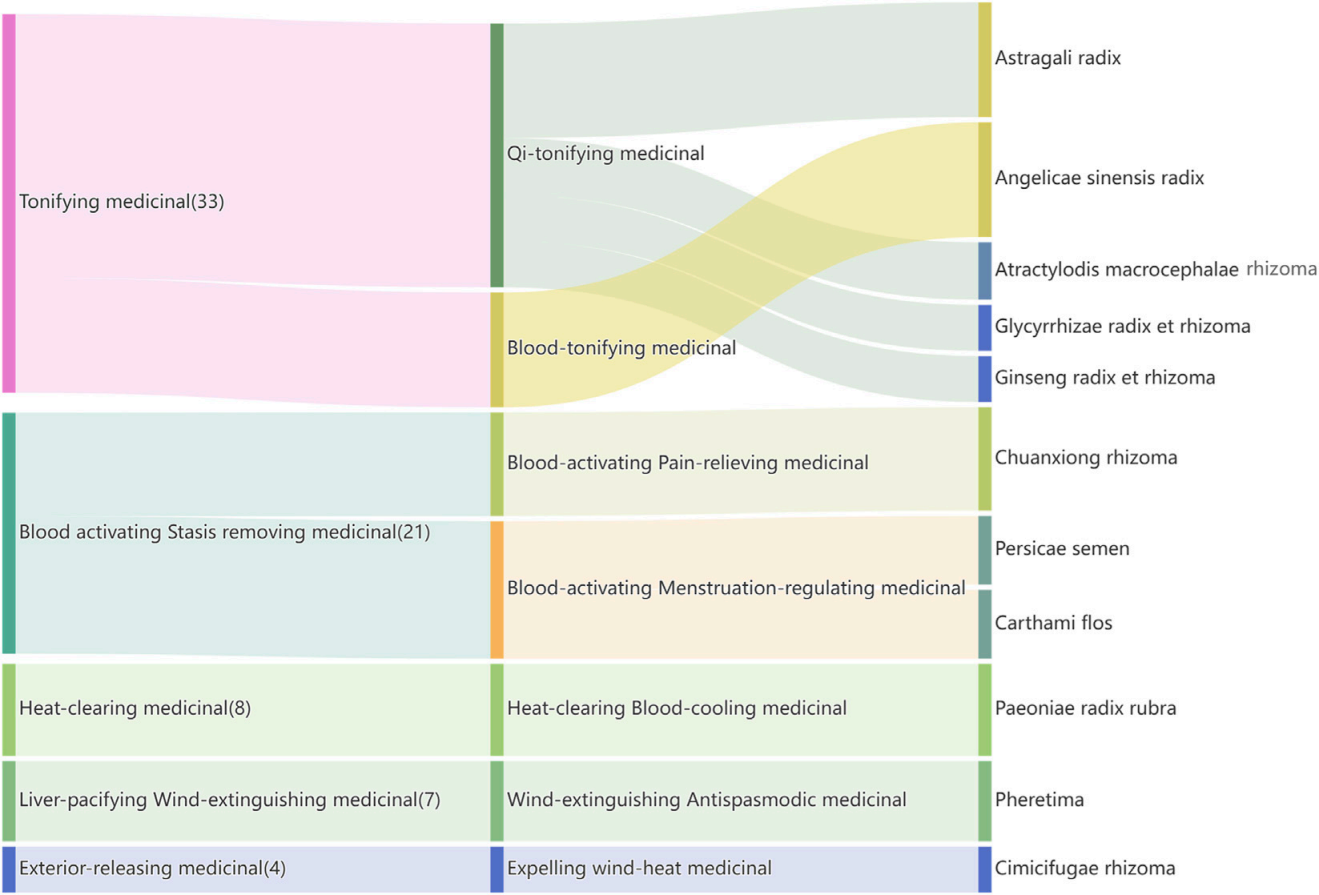
Sankey diagram illustrating the hierarchical categorization of TCM effects of high-frequency Chinese medicinal materials for post-stroke fatigue.

Regarding the TCM therapeutic effects of the Chinese medicinal materials, the classification standard was established according to *Chinese Materia Medica* (National Planned Textbooks) and SymMap (Wu Y. et al., 2019; Zhong and Yang, 2021). The ingredients with a usage frequency exceeding 20% were classified into five categories: tonifying medicinal, blood activating stasis removing medicinal, exterior-releasing medicinal, heat-clearing medicinal, and liver-pacifying wind-extinguishing medicinal. Among these, tonifying medicinal (33 times, 45.21%) and blood activating stasis removing medicinal (21 times, 28.77%) were the two most predominant categories. The distribution flow is visualized via the Sankey diagram in Figure 7.

## 4 Discussion

CHM has gained strong recognition and has been extensively utilized for centuries due to its therapeutic efficacy in treating stroke and its sequalae. However, to date, no study has systematically evaluated the scientific evidence to validate its efficacy for PSF, nor has there been an analysis of the associated medicinal characteristics and prescriptive patterns. In this review, we included 19 trials (1,474 patients) by a well-designed search strategy, integrated meta-analysis with medication pattern profiling to address these gaps.

### 4.1 Efficacy of CHM in relieving fatigue symptom

Given that a single fatigue score will not fully capture the multidimensional impact of this symptom on stroke survivors (English et al., 2024), we utilized the FSS, VAS and FAI as primary outcome measures to evaluate the potential benefits of CHM for PSF (Ozyemisci-Taskiran et al., 2019; Byeon and Lee, 2004; Tseng et al., 2010). Our meta-analysis demonstrated that CHM significantly reduced FSS scores in stroke patients. However, CHM did not significantly reduce VAS scores, likely because merely two studies were incorporated into the analysis. Additionally, the improvement in FAI after CHM treatment was described qualitatively, as the relevant data from the two included studies were ineligible for meta-analysis due to inconsistent reporting formats. While conventional pharmacotherapies for fatigue relief typically target specific biochemical pathways, their precise effects remain elusive due to the complex physiological networks implicated in PSF (Liang et al., 2025). In contrast, CHM offers unique clinical advantages through its multi-target, multi-link, and multi-pathway characteristics, providing integrated regulation based on holistic philosophy (Chow, 2022). CHM was found to exert anti-fatigue effect via multi-target integrated regulation of energy metabolic disturbance, toxic metabolite deposition, oxidative stress imbalance, gut microbial dysbiosis, chronic inflammatory response and neurotransmitter dysregulation, in general, chronic, or disease-related fatigue (Yu et al., 2023). Accordingly, the CHM formulas included in this study demonstrated a positive effect in relieving fatigue symptom in stroke patients. Buyang Huanwu decoction was a frequently used herbal decoction among the included studies, as it is a classic Chinese prescription with a significant clinical reputation. This classic formula has long been applied in China for stroke management and the rehabilitation of post-stroke patients. It exerts multi-pathway neuroprotective effects, including inhibiting inflammatory responses, improving mitochondrial activity, regulating cerebral energy metabolism and modulating gut microbiota (Qin et al., 2025; Zhang et al., 2018). Since inflammation, cellular energy depletion, and gut dysbiosis are key biological factors contributing to fatigue in stroke (English et al., 2024; Li et al., 2022), a meta-analysis has reported the potential effect of Buyang Huanwu decoction in reducing clinical symptoms in patients with PSF (Jin et al., 2021).

Although our meta-analysis suggests promising results for the use of CHM in treating PSF, significant heterogeneity was observed. Consequently, we conducted subgroup analyses based on the type of CHM formulas. The results indicated that heterogeneity was negligible in studies involving Chinese patent medicines, as these are standardized, mass-produced pharmaceutical products approved by the National Medical Products Administration (Zhang et al., 2022). Conversely, the high heterogeneity in other studies likely arises from the inherently personalized nature of Chinese herbal decoctions. These herbal prescriptions are often tailored to patients based on syndrome differentiation, leading to variations in composition (additions and subtractions) and dosages across different studies.

### 4.2 Efficacy of CHM on quality of life, mood and motor functions

PSF patients are generally partially or completely dependent; consequently, they are often unable to meet the demands of an active social and professional life, which significantly impacts their quality of life. Furthermore, PSF may manifest as reduced physical activity and may co-exist with mood disorders (Passier et al., 2011). Physical deconditioning, depression, and anxiety may, in turn, exacerbate PSF symptoms by hindering adequate coping with the consequences of the disease (Staub and Bogousslavsky, 2001; Williams et al., 1999). Therefore, the assessment of quality of life, mood, and motor functions in PSF patients is essential for evaluating their prognosis and long-term health outcomes. Our pooled results provide evidence supporting the effectiveness of CHM as a holistic management strategy for PSF. Specifically, CHM interventions led to significant improvements in quality of life, manifested by increased scores in the SS-QOL-E, BI and MBI. Moreover, reductions in HAMA and HAMD scores further support the role of CHM in alleviating mood disturbances. These findings corroborate existing literature, which has explored the benefits of the single herb Astragali radix and Qi-supplementing Chinese *Materia medica* in improving the aforementioned indicators (Xu et al., 2020; Guo et al., 2012), thereby highlighting the multimodal effects of CHM in PSF treatment.

### 4.3 Medication pattern of CHM for PSF

Although findings from our meta-analysis offer evidence to support the effectiveness of CHM interventions for PSF treatment, the rationale behind the selection of medicinal combinations within these CHM formulas needs to be further explicitly described. Therefore, we conducted a medication pattern analysis to reveal the key Chinese medicinal materials frequently utilized in clinical practice.

In TCM diagnostics, PSF lacks a specific formal designation and is generally grouped under the clinical categories of “lassitude” and “sluggishness”. The clinical manifestations of fatigue bear a close resemblance to “Xu Lao” (Consumptive Disease). Fatigue is a chronic debilitating condition caused by various factors, with its primary pathogenesis involving Zang-fu organ depletion, insufficiency of qi, blood, yin, and yang, as well as the dysfunction of the qi mechanism. This suggests that physical overexertion consumes the body’s qi and blood, damaging the physical form and Zang-fu organs externally; meanwhile, mental overexertion silently depletes essence and blood, injuring the Zang-fu organs internally (Zeng and Tang, 2012). Therefore, from a TCM theoretical perspective, it is hypothesized that deficiency and stagnation patterns are the fundamental pathogenesis of PSF (Ren et al., 2024).

From the medication frequency analysis, it highlights a consistent pattern in the herbal components utilized for PSF therapy. The most frequently used Chinese medicinal material is Astragali radix (Huang Qi), which has the functions of tonifying qi and ascending yang, engendering fluids and nourishing blood, resolving stagnation and unblocking painful obstruction. Its slightly warm nature and sweet fragrance enable it to tonify the spleen and supplement the middle-Jiao qi, making it highly effective for individuals with yang deficiency or qi deficiency (Zhang et al., 2024). Recent meta-analysis has confirmed its anti-fatigue effects among stroke survivors (Xu et al., 2020). The second most frequent Chinese medicinal material is Angelicae sinensis radix (Dang Gui), known for its warm, sweet and pungent, that tonifies and activates blood (Fang et al., 2012). As recorded in *Complete Compendium of Jingyue Materia Medica Orthodoxy*, this herb is “sweet and thick in flavor, thus specializing in nourishing blood; it is light and pungent in qi, hence capable of activating blood circulation. It embodies movement within tonification and tonification within circulation. Truly, it is the qi-regulating herb for blood disorders and the supreme sacred medicinal in blood regulation.” Consequently, Angelicae sinensis radix has broad applications in preventing and managing cerebrovascular conditions, particularly in ameliorating cerebral ischemic damage (Chen et al., 2024). Moreover, the anti-fatigue and mood regulation effects of Angelicae sinensis radix have been well-demonstrated in both exercise-stress and chronic unpredictable stress animal models (Yeh et al., 2014; Shen et al., 2016). The third most frequent Chinese medicinal material is Chuanxiong rhizoma (Chuan Xiong), which activates blood and moves qi, dispels wind and alleviates pain. Chuanxiong rhizoma is pungent and warm in nature with ascending and dispersing properties. It can ascend to the head and is mainly indicated for pain syndromes caused by blood deficiency and qi stagnation. Its therapeutic effects are manifested through multiple pharmacological actions, such as dilating blood vessels, improving microcirculation and anti-cerebral ischemic. The effective material basis mainly includes ligustrazine and volatile oil (Ran et al., 2011; Li et al., 2021).

Analysis of the nature, flavor, and meridian tropism of high-frequency Chinese medicinal materials shows that their predominant medicinal properties are warm and slightly warm, suggesting that the core therapeutic principle for PSF focuses on warming and reinforcing Yang-qi, which is consistent with the disease’s key pathogenesis of deficiency and stagnation. Medicinals with sweet and pungent flavors predominated. The sweet flavor primarily tonifies deficiency, while the pungent flavor promotes the circulation of qi and blood. This reflects the TCM strategy of “tonifying without stagnation” in treating cerebral infarction (Zhou et al., 2015). The medicinal actions mainly target the spleen, liver and heart meridians. The spleen governs transportation, transformation and blood containment; it nourishes the muscles, improves appetite and digestion, and maintains normal mental and emotional activities. The liver regulates qi movement and stores blood, controlling systemic tendon movement and emotional regulation. The heart dominates the blood vessels and houses the spirit, functioning to propel blood circulation and sustain mental activities (Wang and Zhu, 2011). According to TCM theory, these functions are hypothesized to be closely associated with the core pathogenesis of PSF, namely, spleen qi deficiency, liver blood insufficiency, and malnourishment of the heart spirit. Based on this theoretical framework, it is proposed that the use of Chinese medicinal materials targeting and enhancing the functions of these three TCM Zang-organs may help alleviate symptoms in patients with PSF.

The Sankey flow diagram indicates that clinically commonly adopted Chinese medicinal materials for PSF treatment mainly focus on tonification and blood-activating stasis resolution. It indicates that in routine clinical practice, TCM clinicians usually take tonifying deficiency, activating blood circulation and resolving stasis as the core therapeutic principles, and adopt corresponding Chinese medicinal materials to alleviate symptoms of PSF. Meanwhile, personalized strategies based on syndrome differentiation are combined, including clearing heat and cooling blood, calming the liver to extinguish wind, and expelling wind-heat.

### 4.4 Strengths and limitations

In this study, we employed a novel approach to quantitatively explore the use of CHM in PSF treatment for the first time. By integrating meta-analysis with medication pattern analysis, we provide preliminary evidence for the rational application of CHM interventions. Additionally, we identified key Chinese medicinal materials and core TCM therapeutic strategies, offering new insights into the optimal formulation of CHM prescriptions and even the development of functional foods, since the top three high-frequency ingredients are all medicinal and edible homologous (Zhong et al., 2024). These findings can assist clinicians in selecting more effective treatment strategies, improve clinical outcomes, and support the development of related products to benefit a broader population. Moreover, the combined methodology of this study can further facilitate research on other CHM-based treatments, promoting the modernization and standardization of TCM strategies.

Certain limitations were present in this study. Firstly, primary metric such as FSS showed statistically significant heterogeneity in our pooled results. Subgroup analysis revealed that this may be due to the lack of standardization of CHM composition in the experimental group in the included studies. Therefore, future research should focus on the development and application of standardized CHM prescriptions, while reasonably weighing and considering TCM syndrome differentiation in patients with PSF. Furthermore, although the FSS is recommended as the primary outcome measure in post-stroke fatigue research, future trials should not only report statistically significant differences in FSS scores but also consider clinically meaningful change thresholds, such as the Reliable Change Index, to facilitate interpretation of treatment benefits (Barker-Collo et al., 2024). Second, the generalizability of our results may be limited due to the geographic concentration of studies in China. Nevertheless, one of the included studies was published in English (Pan et al., 2016); another English article was excluded during our selection process because it utilized *Ginkgo* in the control group (Liu et al., 2016). Additionally, patients’ treatment preferences contribute to this geographic distribution, as Asian stroke survivors tend to use TCM therapies with high adherence (Lee et al., 2004). Third, most of the included RCTs presented methodological limitations, leading to a moderate overall bias rating, while a few studies were identified as high-risk, primarily due to flaws in randomization procedures and incomplete data reporting. These biases may have influenced the overall quality of the findings to some extent. Therefore, more rigorous study designs and a wider range of participants are essential for future research.

## 5 Conclusion

The present study comprehensively investigated the application of CHM for the management of PSF through meta-analysis and medication pattern analysis. Our meta-analysis showed improvements in relieving fatigue symptom (FSS) and quality of life, mood and motor functions (SS-QOL-E, FMA, BI and MBI, HAMA, HAMD). These results suggest that CHM has meaningful multimodal effects as a holistic therapy. Prominent ingredients such as Astragali radix, Angelicae sinensis radix, Chuanxiong rhizoma were identified as key components in CHM formulations. The overall medicinal profile is highly concentrated on warm or slightly warm natures, sweet and pungent flavors, and a tropism to the spleen, liver, and heart meridians, with core efficacies of tonifying deficiency and activating blood to resolve stasis. These findings provide valuable insights into the rational and effective use of CHM in managing PSF. Further multicenter RCTs featuring larger sample sizes, standardized CHM formulations, and rigorous methodologies are warranted to confirm these results and facilitate the integration of CHM into global clinical practice for PSF.

## Data Availability

The original contributions presented in the study are included in the article/Supplementary Material, further inquiries can be directed to the corresponding author.
