# Supplementary material for: Quantitative analysis of Chinese herbal medicine for post-stroke fatigue: a meta-analysis and medication pattern study
Source: Front Pharmacol. 2026 Jul 31;17:1880065. doi: 10.3389/fphar.2026.1880065 (PMC13473405; doi:10.3389/fphar.2026.1880065)
Supplement: Supplementary file 1 [file Table1.docx]

***Supplementary Material***

**Supplementary Table S1** Results of the PubMed and CNKI search strategy

(Other databases could also follow this format)

| **The search strategy for PubMed** | |
| --- | --- |
| **Nubmer** | **Search terms** |
| #1 | ((((((((((((((((((((((((((Stroke[MeSH Terms]) OR (Hemorrhagic Stroke[MeSH Terms])) OR (Ischemic Stroke[MeSH Terms])) OR (Cerebrovascular Disorders[MeSH Terms])) OR (Brain Ischemia[MeSH Terms])) OR (Brain Infarction[MeSH Terms])) OR (Brain Stem Infarctions[MeSH Terms])) OR (Cerebral Infarction[MeSH Terms])) OR (Infarction, Anterior Cerebral Artery[MeSH Terms])) OR (Infarction, Middle Cerebral Artery[MeSH Terms])) OR (Infarction, Posterior Cerebral Artery[MeSH Terms])) OR (Ischemic Attack, Transient[MeSH Terms])) OR (Cerebral Arterial Diseases[MeSH Terms])) OR (Cerebral Hemorrhage[MeSH Terms])) OR (Intracranial Hemorrhage[MeSH Terms])) OR (Subarachnoid Hemorrhage[MeSH Terms])) OR (Stroke Rehabilitation[MeSH Terms])) OR (Hemiplegia[MeSH Terms])) OR (apoplexy[Title/Abstract])) OR (brain vascular acciden[Title/Abstract])) OR (post-stroke[Title/Abstract])) OR (Cerebrovascular Accident*[Title/Abstract])) OR (Acute Stroke[Title/Abstract])) OR (Cerebrum HemORrhage*[Title/Abstract])) OR (Parenchymal HemORrhage*[Title/Abstract])) OR (Intracerebral HemORrhage*[Title/Abstract])) OR (Brain HemORrhage*[Title/Abstract]) |
|  | **Result:** 506179 |
| #2 | (((((((((((((((Fatigue[MeSH Terms]) OR (Mental Fatigue[MeSH Terms])) OR (Muscle Fatigue[MeSH Terms])) OR (Fatigue Syndrome, Chronic[MeSH Terms])) OR (lethargy[MeSH Terms])) OR (Asthenia[MeSH Terms])) OR (Lethargy[MeSH Terms])) OR (Apathy[MeSH Terms])) OR (weariness[Title/Abstract])) OR (weariness[Title/Abstract])) OR (exhaustion[Title/Abstract])) OR (lassitude[Title/Abstract])) OR (tiredness[Title/Abstract])) OR (listlessness[Title/Abstract])) OR (post-stroke fatigue[Title/Abstract])) OR (PSF[Title/Abstract]) |
|  | **Result:** 106320 |
| #3 | (((((((((((traditional Chinese medicine[MeSH Terms]) OR (Herbal Medicine[MeSH Terms])) OR (Chinese Herbal Drugs[MeSH Terms])) OR (Medicine, Traditional[MeSH Terms])) OR (plant preparations[MeSH Terms])) OR (Medicine, East Asian Traditional[MeSH Terms])) OR (Chinese patent medicine[Title/Abstract])) OR (Herbal Medicine[Title/Abstract])) OR (Ethnomedicine[Title/Abstract])) OR (Indigenous Medicine[Title/Abstract])) OR (Medicine, Folk[Title/Abstract])) OR (Folk Remedy[Title/Abstract]) |
|  | **Result:** 356405 |
| #4 | #1 AND #2 AND #3 |
|  | **Result:** 13 |
| **The search strategy for CNKI** | |
| ( SU=卒中 OR SU=中风 OR SU=脑卒中 OR SU=脑血管意外 OR SU=缺血性中风 OR SU=出血性中风 OR SU=脑梗死 OR SU=脑梗塞 OR SU=脑栓塞 OR SU=脑出血 OR SU=蛛网膜下腔出血 ) AND ( SU=疲劳 OR SU=慢性疲劳 OR SU=疲劳综合症 OR SU=精神疲劳 OR SU=肌肉疲劳 OR SU=疲倦 OR SU=乏力 OR SU=虚弱 OR SU=卒中后疲劳) AND (SU=中西医 OR SU = 中医 OR SU = 中医药 OR SU = 中药 OR SU = 中成药 OR SU = 中草药 OR SU = 草药 OR SU = 方药 OR SU = 复方 OR SU = 汤OR SU = 片OR SU = 饮片 OR SU = 丸 OR SU = 散 OR SU = 胶囊 OR SU = 颗粒 OR SU = 配方颗粒 OR SU = 液 OR SU = 合剂 OR SU = 水OR SU = 膏OR SU = 冲剂 OR SU = 丹 OR SU = 酒 OR SU = 茶 OR SU = 露 )  **Result:** 130 | |

**Supplementary Table S2** Composition of the CHM formulas of the included studies

| **Study ID** | **CHM intervention** | **Formulation** | **Components** | **Taxonomic validation** | **ConPhyMP-informed assessment** |
| --- | --- | --- | --- | --- | --- |
| Li 2023 | Self-formulated herbal decoction | Chinese herbal decoction | Astragali radix 80g, Angelicae sinensis radix 15g, Mori Ramulus 15g, Spatholobi Caulis 15g, Paeoniae radix rubra 15g, Pheretima 15g, Codonopsis Radix 15g, Cyathulae Radix 15g, Persicae semen 10g, Cimicifugae rhizoma 10g, Chuanxiong rhizoma 10g, Atractylodis macrocephalae rhizoma 10g, Carthami Flos 5g, Citri Reticulatae Pericarpium 5g | Astragalus membranaceus（Fisch.）Bge. [Fabaceae; Astragali radix], Angelica sinensis（Oliv.）Diels[Apiaceae; Angelicae sinensis radix],  *Morus alba* L. **[**Moraceae; Mori Ramulus],  *Spatholobus suberectus* Dunn [Fabaceae; Spatholobi Caulis],  Paeonia lactiflora Pall. [Paeoniaceae; Paeoniae radix rubra],  Pheretima aspergillum（E. Perrier）[Megascolecidae; Pheretima],  *Codonopsis pilosula* (Franch.) Nannf. [Campanulaceae; Codonopsis Radix],  *Cyathula officinalis* Kuan [Amaranthaceae; Cyathulae Radix],  Prunus persica（L.）Batsch [Rosaceae; Persicae semen],  Cimicifuga foetida L. [Ranunculaceae; Cimicifugae rhizoma],  Ligusticum chuanxiong Hort. [Apiaceae; Chuanxiong rhizoma],  Atractylodes macrocephala Koidz. [Asteraceae; Atractylodis macrocephalae rhizoma],  *Carthamus tinctorius* L. [Asteraceae; Carthami Flos],  *Citrus reticulata* Blanco [Rutaceae; Citri Reticulatae Pericarpium] | Adequate |
| Liang 2016 | Buyang Huanwu Decoction combined with Modified Xiaoyao San | Chinese herbal decoction | Astragali radix 50g, Angelicae sinensis radix 10g, Chuanxiong rhizoma 10g, Paeoniae radix rubra 15g, Paeoniae radix Alba 10g, Pheretima 10g, Persicae Semen 10g, Carthami flos 10g, Bupleuri Radix 10g, Atractylodis Macrocephalae Rhizoma 10g, Poria 10g, Glycyrrhizae radix et rhizoma 6g, Menthae Haplocalycis Herba 3g, Zingiberis Rhizoma Recens 3g | Astragalus membranaceus（Fisch.）Bge. [Fabaceae; Astragali radix], Angelica sinensis（Oliv.）Diels[Apiaceae; Angelicae sinensis radix],  Ligusticum chuanxiong Hort. [Apiaceae; Chuanxiong rhizoma],  Paeonia lactiflora Pall. [Paeoniaceae; Paeoniae radix rubra],  Paeonia lactiflora Pall. [Paeoniaceae; Paeoniae radix Alba],  Pheretima aspergillum（E. Perrier）[Megascolecidae; Pheretima],  Prunus persica（L.）Batsch [Rosaceae; Persicae semen],  Carthamus tinctorius L. [Asteraceae; Carthami flos],  *Bupleurum chinense* DC. [Apiaceae; Bupleuri Radix],  *Atractylodes macrocephala* Koidz. [Asteraceae; Atractylodis Macrocephalae Rhizoma],  *Poria cocos*（Schw.）Wolf [Polyporaceae; Poria],  Glycyrrhiza uralensis Fisch. [Fabaceae; Glycyrrhizae radix et rhizoma],  *Mentha haplocalyx* Briq. [ Lamiaceae; Menthae Haplocalycis Herba],  *Zingiber officinale* Roscoe [Zingiberaceae; Zingiberis Rhizoma Recens] | Adequate |
| Ye 2018 | Buyang Huanwu Decoction | Chinese herbal decoction | Astragali radix 120g, Paeoniae radix rubra 15g, Angelicae sinensis radix 10g, Chuanxiong rhizoma 10g, Persicae Semen 10g, Carthami flos 10g, Pheretima 10g | Astragalus membranaceus（Fisch.）Bge. [Fabaceae; Astragali radix], Paeonia lactiflora Pall. [Paeoniaceae; Paeoniae radix rubra],  Angelica sinensis（Oliv.）Diels[Apiaceae; Angelicae sinensis radix],  Ligusticum chuanxiong Hort. [Apiaceae; Chuanxiong rhizoma],  Prunus persica（L.）Batsch [Rosaceae; Persicae semen],  Carthamus tinctorius L. [Asteraceae; Carthami flos],  Pheretima aspergillum（E. Perrier）[Megascolecidae; Pheretima] | Adequate |
| Wen 2013 | Modified Buyang Huanwu Decoction | Chinese herbal decoction | Astragali radix 60g, Angelicae sinensis radix 10g, Chuanxiong rhizoma 10g, Paeoniae radix rubra 15g, Pheretima 6g, Persicae Semen 15g, Carthami flos 15g, Atractylodis Macrocephalae Rhizoma 15g, Dioscoreae Rhizoma 30g, Rehmanniae Radix 30g, Morindae Officinalis Radix 15g, Eucommiae Cortex 30g, Amomi Fructus 10g | Astragalus membranaceus（Fisch.）Bge. [Fabaceae; Astragali radix], Angelica sinensis（Oliv.）Diels[Apiaceae; Angelicae sinensis radix],  Ligusticum chuanxiong Hort. [Apiaceae; Chuanxiong rhizoma],  Paeonia lactiflora Pall. [Paeoniaceae; Paeoniae radix rubra],  Pheretima aspergillum（E. Perrier）[Megascolecidae; Pheretima],  Prunus persica（L.）Batsch [Rosaceae; Persicae semen],  Carthamus tinctorius L. [Asteraceae; Carthami flos],  *Atractylodes macrocephala* Koidz. [Asteraceae; Atractylodis Macrocephalae Rhizoma],  *Dioscorea opposita* Thunb. [Dioscoreaceae; Dioscoreae Rhizoma], *Rehmannia glutinosa* Libosch. [Orobanchaceae; Rehmanniae Radix], *Morinda officinalis* F.C.How [Rubiaceae; Morindae Officinalis Radix],  *Eucommia ulmoides* Oliv. [Eucommiaceae; Eucommiae Cortex], *Amomum villosum* Lour. [Zingiberaceae; Amomi Fructus] | Adequate |
| Ye 2016 | Modified Buyang Huanwu Decoction | Chinese herbal decoction | Astragali radix 120g, Angelicae sinensis radix 10g, Chuanxiong rhizoma 10g, Paeoniae radix rubra 15g, Pheretima 10g | Astragalus membranaceus（Fisch.）Bge. [Fabaceae; Astragali radix], Angelica sinensis（Oliv.）Diels[Apiaceae; Angelicae sinensis radix],  Ligusticum chuanxiong Hort. [Apiaceae; Chuanxiong rhizoma],  Paeonia lactiflora Pall. [Paeoniaceae; Paeoniae radix rubra],  Pheretima aspergillum（E. Perrier）[Megascolecidae; Pheretima] | Partially adequate |
| Lin 2020 | Buzhong Yiqi Decoction | Chinese herbal decoction | Astragali radix 30g, Codonopsis Radix 15g, Cimicifugae rhizoma 3g, Bupleuri Radix 6g, Glycyrrhizae radix et rhizoma 3g, Atractylodis macrocephalae rhizoma 10g, Angelicae sinensis radix 10g, Citri Reticulatae Pericarpium 10g | Astragalus membranaceus（Fisch.）Bge. [Fabaceae; Astragali radix], *Codonopsis pilosula* (Franch.) Nannf. [Campanulaceae; Codonopsis Radix], Cimicifuga foetida L. [Ranunculaceae; Cimicifugae rhizoma],  *Bupleurum chinense* DC. [Apiaceae; Bupleuri Radix],  Glycyrrhiza uralensis Fisch. [Fabaceae; Glycyrrhizae radix et rhizoma],  Atractylodes macrocephala Koidz. [Asteraceae; Atractylodis macrocephalae rhizoma],  Angelica sinensis（Oliv.）Diels[Apiaceae; Angelicae sinensis radix],  *Citrus reticulata* Blanco [Rutaceae; Citri Reticulatae Pericarpium] | Partially adequate |
| Feng 2014 | Buzhong Yiqi Decoction | Chinese herbal decoction | Astragali radix 30g, Codonopsis Radix 15g, Cimicifugae rhizoma 3g, Bupleuri Radix 6g, Glycyrrhizae radix et rhizoma 3g, Atractylodis Macrocephalae Rhizoma 10g, Angelicae sinensis radix 10g, Citri Reticulatae Pericarpium 10g | Astragalus membranaceus（Fisch.）Bge. [Fabaceae; Astragali radix], *Codonopsis pilosula* (Franch.) Nannf. [Campanulaceae; Codonopsis Radix], Cimicifuga foetida L. [Ranunculaceae; Cimicifugae rhizoma],  *Bupleurum chinense* DC. [Apiaceae; Bupleuri Radix],  Glycyrrhiza uralensis Fisch. [Fabaceae; Glycyrrhizae radix et rhizoma], *Atractylodes macrocephala* Koidz. [Asteraceae; Atractylodis Macrocephalae Rhizoma],  Angelica sinensis（Oliv.）Diels[Apiaceae; Angelicae sinensis radix],  *Citrus reticulata* Blanco [Rutaceae; Citri Reticulatae Pericarpium] | Adequate |
| Sima 2017 | Buzhong Yiqi Decoction | Chinese herbal decoction | Astragali radix 30g, Codonopsis Radix 15g, Cimicifugae rhizoma 3g, Bupleuri Radix 6g, Glycyrrhizae radix et rhizoma 3g, Atractylodis macrocephalae rhizoma 10g, Angelicae sinensis radix 10g, Citri Reticulatae Pericarpium 10g | Astragalus membranaceus（Fisch.）Bge. [Fabaceae; Astragali radix], *Codonopsis pilosula* (Franch.) Nannf. [Campanulaceae; Codonopsis Radix], Cimicifuga foetida L. [Ranunculaceae; Cimicifugae rhizoma],  *Bupleurum chinense* DC. [Apiaceae; Bupleuri Radix],  *Glycyrrhiza uralensis* Fisch. [Fabaceae; Glycyrrhizae radix et rhizoma], Atractylodes macrocephala Koidz. [Asteraceae; Atractylodis macrocephalae rhizoma],  Angelica sinensis（Oliv.）Diels[Apiaceae; Angelicae sinensis radix],  *Citrus reticulata* Blanco [Rutaceae; Citri Reticulatae Pericarpium] | Adequate |
| Sun 2014 | Dabu Gan Decoction | Chinese herbal decoction | Cinnamomi ramulus 15g, Zingiberis Rhizoma Recens 15g, Schisandrae Chinensis Fructus 15g, Inulae Flos 5g, Moutan Cortex 5g, Lophatheri Herba 5g, Dioscoreae Rhizoma 15g | *Cinnamomum cassia* (L.) J.Presl [Lauraceae; Cinnamomi ramulus], *Zingiber officinale* Roscoe [Zingiberaceae; Zingiberis Rhizoma Recens],  *Schisandra chinensis* (Turcz.) Baill. [Schisandraceae; Schisandrae Chinensis Fructus],  *Inula britannica* L. [Asteraceae; Inulae Flos],  *Paeonia × suffruticosa* Andrews [Paeoniaceae; Moutan Cortex], *Lophatherum gracile* Brongn. [Poaceae; Lophatheri Herba],  *Dioscorea opposita* Thunb. [Dioscoreaceae; Dioscoreae Rhizoma] | Partially adequate |
| Wu 2019 | Erigeron Breviscapus Capsule | Chinese patent medicine  (Yunnan Haobang Pharmaceutical Co., Ltd. National Medicine Permission Number: Z53021653) | Erigerontis Herba | *Erigeron breviscapus*（Vant.）Hand.-Mazz. [Asteraceae; Erigerontis Herba] | Adequate |
| Sun 2018 | Modified Buyang Huanwu Decoction | Chinese herbal decoction | Astragali radix 80g, Chuanxiong rhizoma 15g, Paeoniae radix rubra 15g, Achyranthis Bidentatae Radix 15g | Astragalus membranaceus（Fisch.）Bge. [Fabaceae; Astragali radix], Ligusticum chuanxiong Hort. [Apiaceae; Chuanxiong rhizoma],  Paeonia lactiflora Pall. [Paeoniaceae; Paeoniae radix rubra],  *Achyranthes bidentata* Blume [Amaranthaceae; Achyranthis Bidentatae Radix] | Partially adequate |
| He 2017 | Shengmai Injection | Chinese patent medicine  (Shanxi Taihang Pharmaceutical Co., Ltd.  National Medicine Permission Number: Z14020812) | Ginseng radix et rhizoma, Schisandrae Chinensis Fructus, Ophiopogonis Radix | Panax ginseng C. A. Mey. [Araliaceae; Ginseng radix et rhizoma],  *Schisandra chinensis* (Turcz.) Baill. [Schisandraceae; Schisandrae Chinensis Fructus],  *Ophiopogon japonicus* (Thunb.) Ker Gawl. [Asparagaceae; Ophiopogonis Radix] | Adequate |
| Niu 2013 | Xiaopiling Granule | Chinese patent medicine  (Zhejiang Dade Pharmaceutical Co., Ltd.  National Medicine Permission Number: Z19993332) | Ginseng radix et rhizoma, Ophiopogonis Radix, Schisandrae Chinensis Fructus, Astragali radix, Angelicae sinensis radix, Longan Arillus, Cinnamomi Cortex, Ganoderma, Spatholobi Caulis, Poria, Crataegi Fructus, Salviae Miltiorrhizae Radix et Rhizoma, Ziziphi Spinosae Semen, Asini Corii Colla | Panax ginseng C. A. Mey. [Araliaceae; Ginseng radix et rhizoma],  *Ophiopogon japonicus* (Thunb.) Ker Gawl. [Asparagaceae; Ophiopogonis Radix],  *Schisandra chinensis* (Turcz.) Baill. [Schisandraceae; Schisandrae Chinensis Fructus],  Astragalus membranaceus（Fisch.）Bge. [Fabaceae; Astragali radix], Angelica sinensis（Oliv.）Diels[Apiaceae; Angelicae sinensis radix],  *Dimocarpus longan* Lour. [Sapindaceae; Longan Arillus],  *Cinnamomum cassia* (L.) J.Presl [Lauraceae; Cinnamomi Cortex], *Ganoderma lucidum*（Leyss. ex Fr.）Karst. [Ganodermataceae; Ganoderma], *Spatholobus suberectus* Dunn [Fabaceae; Spatholobi Caulis],  *Poria cocos*（Schw.）Wolf [Polyporaceae; Poria],  *Crataegus pinnatifida* Bunge [Rosaceae; Crataegi Fructus],  *Salvia miltiorrhiza* Bunge [Lamiaceae; Salviae Miltiorrhizae Radix et Rhizoma],  *Ziziphus jujuba* Mill.[ Rhamnaceae; Ziziphi Spinosae Semen],  *Equus asinus* Linnaeus [Equidae; Asini Corii Colla] | Adequate |
| Zhang 2024 | Xiao Xu Ming Decoction | Chinese herbal decoction | Ephedrae Herba 9g, Stephaniae Tetrandrae Radix 10g, Ginseng Radix et Rhizoma 10g, Cinnamomi ramulus 9g, Paeoniae Radix Alba 15g, Chuanxiong rhizoma 10g, Glycyrrhizae radix et rhizoma 9g, Armeniacae Semen Amarum 10g, Saposhnikoviae Radix 10g, Scutellariae Radix 9g, Jujubae Fructus 3 pieces, Aconiti Lateralis Radix Praeparata 10g, Zingiberis Rhizoma Recens 3 slices | *Ephedra sinica* Stapf [Ephedraceae; Ephedrae Herba], *Stephania tetrandra* S.Moore [Menispermaceae; Stephaniae Tetrandrae Radix],  *Panax ginseng* C.A.Mey. [Araliaceae; Ginseng Radix et Rhizoma],  *Cinnamomum cassia* Presl [Lauraceae; Cinnamomi ramulus], Paeonia lactiflora Pall. [Paeoniaceae; Paeoniae radix Alba],  Ligusticum chuanxiong Hort. [Apiaceae; Chuanxiong rhizoma],  Glycyrrhiza uralensis Fisch. [Fabaceae; Glycyrrhizae radix et rhizoma],  *Prunus armeniaca* L. [Rosaceae; Armeniacae Semen Amarum], *Saposhnikovia divaricata* (Turcz. ex Ledeb.) Schischk. [Apiaceae; Saposhnikoviae Radix],  *Scutellaria baicalensis* Georgi [Lamiaceae; Scutellariae Radix], *Ziziphus jujuba* Mill. [Rhamnaceae; Jujubae Fructus],  *Aconitum carmichaelii* Debeaux [Ranunculaceae; Aconiti Lateralis Radix Praeparata],  *Zingiber officinale* Roscoe [Zingiberaceae; Zingiberis Rhizoma Recens] | Adequate |
| Yang 2020 | Yiqi Fuyang Decoction | Chinese herbal decoction | Rhodiolae Crenulatae Radix et Rhizoma 15g, Morindae Officinalis Radix 12g, Epimedii Folium 12g, Ginseng radix et rhizoma 12g, Bupleuri Radix 6g, Cimicifugae rhizoma 6g, Angelicae sinensis radix 20g, Glycyrrhizae radix et rhizoma 6g | *Rhodiola crenulata* (Hook.f. & Thomson) H.Ohba [Crassulaceae; Rhodiolae Crenulatae Radix et Rhizoma],  *Morinda officinalis* F.C.How [Rubiaceae; Morindae Officinalis Radix],  *Epimedium brevicornu* Maxim. [Berberidaceae; Epimedii Folium], *Panax ginseng* C. A. Mey. [Araliaceae; Ginseng radix et rhizoma],  *Bupleurum chinense* DC. [Apiaceae; Bupleuri Radix], *Cimicifuga foetida* L. [Ranunculaceae; Cimicifugae rhizoma],  *An*g*elica sinensis*（Oliv. ）Diels[Apiaceae; Angelicae sinensis radix],  *Glycyrrhiza uralensis* Fisch. [Fabaceae; Glycyrrhizae radix et rhizoma] | Adequate |
| Tan 2014 | Yulang Xiaopi Decoction | Chinese herbal decoction | Astragali radix 30g, Millettiae Pulchrae Radix 20g, Rehmanniae Radix 15g, Persicae Semen 15g, Pheretima 10g, Acori Tatarinowii Rhizoma 10g, Angelicae sinensis radix 10g, Paeoniae radix rubra 10g, Carthami flos 10g, Chuanxiong rhizoma 5g | Astragalus membranaceus（Fisch.）Bge. [Fabaceae; Astragali radix], *Millettia pulchra* (Voigt) Kurz [Fabaceae; Millettiae Pulchrae Radix], *Rehmannia glutinosa* (Gaertn.) Libosch. ex DC.[Orobanchaceae; Rehmanniae Radix],  Prunus persica（L.）Batsch [Rosaceae; Persicae semen],  *Pheretima aspergillum*（E. Perrier）[Megascolecidae; Pheretima],  *Acorus tatarinowii* Schott [Acoraceae; Acori Tatarinowii Rhizoma],  *An*g*elica sinensis*（Oliv.）Diels[Apiaceae; Angelicae sinensis radix],  *Paeonia lactiflora* Pall. [Paeoniaceae; Paeoniae radix rubra],  *Carthamus tinctorius* L. [Asteraceae; Carthami flos],  *Ligusticum chuanxiong* Hort. [Apiaceae; Chuanxiong rhizoma] | Partially adequate |
| Jiang 2012 | Shuangbu Decoction | Chinese herbal decoction | Astragali radix 45-120g, Codonopsis Radix 15g, Atractylodis macrocephalae rhizoma 10g, Cimicifugae rhizoma 10g, Angelicae sinensis radix 15g, Paeoniae radix rubra 15g, Chuanxiong rhizoma 10g, Persicae Semen 10g, Carthami flos 5g, Pheretima 15g, Spatholobi Caulis 15g, Cyathulae Radix 15g, Mori Ramulus 15g, Citri Reticulatae Pericarpium 6g | Astragalus membranaceus（Fisch.）Bge. [Fabaceae; Astragali radix], *Codonopsis pilosula* (Franch.) Nannf. [Campanulaceae; Codonopsis Radix], Atractylodes macrocephala Koidz. [Asteraceae; Atractylodis macrocephalae rhizoma],  Cimicifuga foetida L. [Ranunculaceae; Cimicifugae rhizoma],  Angelica sinensis（Oliv.）Diels[Apiaceae; Angelicae sinensis radix],  Paeonia lactiflora Pall. [Paeoniaceae; Paeoniae radix rubra],  Ligusticum chuanxiong Hort. [Apiaceae; Chuanxiong rhizoma],  Prunus persica（L.）Batsch [Rosaceae; Persicae semen],  Carthamus tinctorius L. [Asteraceae; Carthami flos],  Pheretima aspergillum（E. Perrier）[Megascolecidae; Pheretima], *Spatholobus suberectus* Dunn [Fabaceae; Spatholobi Caulis],  *Cyathula officinalis* Kuan [Amaranthaceae; Cyathulae Radix], *Morus alba* L. **[**Moraceae; Mori Ramulus],  *Citrus reticulata* Blanco [Rutaceae; Citri Reticulatae Pericarpium] | Partially adequate |
| Liang 2013 | Yulang Xiaopi Decoction | Chinese herbal decoction | Astragali radix 30g, Millettiae Pulchrae Radix 20g, Rehmanniae Radix 15g, Persicae Semen 15g, Pheretima 10g, Acori Tatarinowii Rhizoma 10g, Angelicae sinensis radix 10g, Paeoniae radix rubra 10g, Carthami flos 10g, Chuanxiong rhizoma 5g | Astragalus membranaceus（Fisch.）Bge. [Fabaceae; Astragali radix], *Millettia pulchra* (Voigt) Kurz [Fabaceae; Millettiae Pulchrae Radix], *Rehmannia glutinosa* (Gaertn.) Libosch. ex DC.[Orobanchaceae; Rehmanniae Radix],  Prunus persica（L.）Batsch [Rosaceae; Persicae semen],  Pheretima aspergillum（E. Perrier）[Megascolecidae; Pheretima],  *Acorus tatarinowii* Schott [Acoraceae; Acori Tatarinowii Rhizoma],  Angelica sinensis（Oliv.）Diels[Apiaceae; Angelicae sinensis radix],  Paeonia lactiflora Pall. [Paeoniaceae; Paeoniae radix rubra],  Carthamus tinctorius L. [Asteraceae; Carthami flos],  Ligusticum chuanxiong Hort. [Apiaceae; Chuanxiong rhizoma] | Adequate |
| Pan 2016 | Buyang Huanwu Decoction | Chinese herbal decoction | Astragali radix, Angelicae sinensis radix, Paeoniae radix rubra, Chuanxiong rhizoma, Persicae Semen, Carthami flos, Pheretima | Astragalus membranaceus（Fisch.）Bge. [Fabaceae; Astragali radix], Angelica sinensis（Oliv.）Diels[Apiaceae; Angelicae sinensis radix],  Paeonia lactiflora Pall. [Paeoniaceae; Paeoniae radix rubra],  Ligusticum chuanxiong Hort. [Apiaceae; Chuanxiong rhizoma],  Prunus persica（L.）Batsch [Rosaceae; Persicae semen],  Carthamus tinctorius L. [Asteraceae; Carthami flos],  Pheretima aspergillum（E. Perrier）[Megascolecidae; Pheretima] | Partially adequate |

*Note*: Abbreviations: CHM, Chinese herbal medicine; ConPhyMP, Consensus statement on the Phytochemical Characterisation of Medicinal Plant extracts.

ConPhyMP-informed assessment: The reporting adequacy of each CHM intervention was evaluated according to the reproducibility principles outlined in ConPhyMP ( <https://doi.org/10.3389/fphar.2022.953205> ). This assessment is intended to indicate whether sufficient information was provided to reasonably reproduce the intervention.

**Supplementary Figure S1** Risk of bias assessment for each included study

**Supplementary Figure S2** Funnel plot of FSS

**Supplementary Table S3 GRADE certainty of evidence**

| **Certainty assessment** | | | | | | | **№ of patients** | | **Effect** | **Certainty** |
| --- | --- | --- | --- | --- | --- | --- | --- | --- | --- | --- |
| **№ of studies** | **Study design** | **Risk of bias** | **Inconsistency** | **Indirectness** | **Imprecision** | **Other considerations** | **CHM** | **conventional therapy** | **Absolute (95% CI)** |  |
| FSS | | | | | | | | | | |
| 16 | randomised trials | serious | not serious | not serious | not serious | none | 608 | 615 | MD **7.64 lower** (11.21 lower to 4.07 lower) | ⨁⨁⨁◯ Moderate |
| VAS | | | | | | | | | | |
| 2 | randomised trials | serious | serious | not serious | serious | none | 73 | 71 | MD **1.72 lower** (4 lower to 0.56 higher) | ⨁◯◯◯ Very low |
| SS-QOL-E | | | | | | | | | | |
| 5 | randomised trials | serious | not serious | not serious | not serious | none | 203 | 202 | MD **3.3 higher** (1.44 higher to 5.15 higher) | ⨁⨁⨁◯ Moderate |
| BI | | | | | | | | | | |
| 3 | randomised trials | serious | not serious | not serious | not serious | none | 168 | 168 | MD **12.59 higher** (2.75 higher to 22.44 higher) | ⨁⨁⨁◯ Moderate |
| MBI | | | | | | | | | | |
| 4 | randomised trials | serious | not serious | not serious | not serious | none | 172 | 178 | MD **5.75 higher** (4.28 higher to 7.21 higher) | ⨁⨁⨁◯ Moderate |
| FMA | | | | | | | | | | |
| 3 | randomised trials | serious | not serious | not serious | not serious | none | 129 | 130 | MD **10.42 higher** (2.89 higher to 17.94 higher) | ⨁⨁⨁◯ Moderate |
| HAMA | | | | | | | | | | |
| 2 | randomised trials | serious | not serious | not serious | serious | none | 71 | 71 | MD **1.88 lower** (3.1 lower to 0.66 lower) | ⨁⨁◯◯ Low |
| HAMD | | | | | | | | | | |
| 3 | randomised trials | serious | not serious | not serious | not serious | none | 109 | 116 | MD **6.94 lower**  (10.46 lower to 3.42 lower) | ⨁⨁⨁◯ Moderate |

*Note:* **CI,** confidence interval; **MD,** mean difference; **CT**, conventional therapy; **CHM**, Chinese herbal medicine.

**PRISMA 2020 Checklist**

| **Section and Topic** | **Item #** | **Checklist item** | **Location where item is reported** |
| --- | --- | --- | --- |
| **TITLE** | | |  |
| Title | 1 | Identify the report as a systematic review. | Title |
| **ABSTRACT** | | |  |
| Abstract | 2 | See the PRISMA 2020 for Abstracts checklist. | Abstract |
| **INTRODUCTION** | | |  |
| Rationale | 3 | Describe the rationale for the review in the context of existing knowledge. | 1.Introduction |
| Objectives | 4 | Provide an explicit statement of the objective(s) or question(s) the review addresses. | 1.Introduction |
| **METHODS** | | |  |
| Eligibility criteria | 5 | Specify the inclusion and exclusion criteria for the review and how studies were grouped for the syntheses. | 2.2 Inclusion and Exclusion Criteria |
| Information sources | 6 | Specify all databases, registers, websites, organisations, reference lists and other sources searched or consulted to identify studies. Specify the date when each source was last searched or consulted. | 2.1 Search Strategy |
| Search strategy | 7 | Present the full search strategies for all databases, registers and websites, including any filters and limits used. | 2.1 Search Strategy, Table S1 |
| Selection process | 8 | Specify the methods used to decide whether a study met the inclusion criteria of the review, including how many reviewers screened each record and each report retrieved, whether they worked independently, and if applicable, details of automation tools used in the process. | 2.3 Study Selection and Data Extraction |
| Data collection process | 9 | Specify the methods used to collect data from reports, including how many reviewers collected data from each report, whether they worked independently, any processes for obtaining or confirming data from study investigators, and if applicable, details of automation tools used in the process. | 2.3 Study Selection and Data Extraction |
| Data items | 10a | List and define all outcomes for which data were sought. Specify whether all results that were compatible with each outcome domain in each study were sought (e.g. for all measures, time points, analyses), and if not, the methods used to decide which results to collect. | 2.3 Study Selection and Data Extraction |
|  | 10b | List and define all other variables for which data were sought (e.g. participant and intervention characteristics, funding sources). Describe any assumptions made about any missing or unclear information. | 2.3 Study Selection and Data Extraction |
| Study risk of bias assessment | 11 | Specify the methods used to assess risk of bias in the included studies, including details of the tool(s) used, how many reviewers assessed each study and whether they worked independently, and if applicable, details of automation tools used in the process. | 2.4 Methodological Quality Assessment |
| Effect measures | 12 | Specify for each outcome the effect measure(s) (e.g. risk ratio, mean difference) used in the synthesis or presentation of results. | 2.5 Statistical Analysis |
| Synthesis methods | 13a | Describe the processes used to decide which studies were eligible for each synthesis (e.g. tabulating the study intervention characteristics and comparing against the planned groups for each synthesis (item #5)). | 2.5 Statistical Analysis |
|  | 13b | Describe any methods required to prepare the data for presentation or synthesis, such as handling of missing summary statistics, or data conversions. | 2.5 Statistical Analysis |
|  | 13c | Describe any methods used to tabulate or visually display results of individual studies and syntheses. | 2.5 Statistical Analysis |
|  | 13d | Describe any methods used to synthesize results and provide a rationale for the choice(s). If meta-analysis was performed, describe the model(s), method(s) to identify the presence and extent of statistical heterogeneity, and software package(s) used. | 2.5 Statistical Analysis |
|  | 13e | Describe any methods used to explore possible causes of heterogeneity among study results (e.g. subgroup analysis, meta-regression). | 2.5 Statistical Analysis |
|  | 13f | Describe any sensitivity analyses conducted to assess robustness of the synthesized results. | 2.5 Statistical Analysis |
| Reporting bias assessment | 14 | Describe any methods used to assess risk of bias due to missing results in a synthesis (arising from reporting biases). | 2.5 Statistical Analysis |
| Certainty assessment | 15 | Describe any methods used to assess certainty (or confidence) in the body of evidence for an outcome. | 2.6 Certainty of evidence assessment |
| **RESULTS** | | |  |
| Study selection | 16a | Describe the results of the search and selection process, from the number of records identified in the search to the number of studies included in the review, ideally using a flow diagram. | 3.1 Study Selection, Figure 1 |
|  | 16b | Cite studies that might appear to meet the inclusion criteria, but which were excluded, and explain why they were excluded. | 3.1 Study Selection |
| Study characteristics | 17 | Cite each included study and present its characteristics. | 3.2 Study Characteristics, Table1, Table S2 |
| Risk of bias in studies | 18 | Present assessments of risk of bias for each included study. | 3.3 Risk of bias, Figure2,FigureS1 |
| Results of individual studies | 19 | For all outcomes, present, for each study: (a) summary statistics for each group (where appropriate) and (b) an effect estimate and its precision (e.g. confidence/credible interval), ideally using structured tables or plots. | Figure 3-5 |
| Results of syntheses | 20a | For each synthesis, briefly summarise the characteristics and risk of bias among contributing studies. | 3.4 Effect of CHM on fatigue, 3.5 Effect of CHM on quality of life, 3.6 Effect of CHM on mood and motor functions, Figure 3-5 |
|  | 20b | Present results of all statistical syntheses conducted. If meta-analysis was done, present for each the summary estimate and its precision (e.g. confidence/credible interval) and measures of statistical heterogeneity. If comparing groups, describe the direction of the effect. | 3.4 Effect of CHM on fatigue, 3.5 Effect of CHM on quality of life, 3.6 Effect of CHM on mood and motor functions, Figure 3-5 |
|  | 20c | Present results of all investigations of possible causes of heterogeneity among study results. | 3.4 Effect of CHM on fatigue, 3.5 Effect of CHM on quality of life, 3.6 Effect of CHM on mood and motor functions, Figure 3-5 |
|  | 20d | Present results of all sensitivity analyses conducted to assess the robustness of the synthesized results. | 3.5 Effect of CHM on quality of life, 3.6 Effect of CHM on mood and motor functions, Figure 3-5 |
| Reporting biases | 21 | Present assessments of risk of bias due to missing results (arising from reporting biases) for each synthesis assessed. | 3.7 Publication bias ,  Figure S2 |
| Certainty of evidence | 22 | Present assessments of certainty (or confidence) in the body of evidence for each outcome assessed. | Table S3 |
| **DISCUSSION** | | |  |
| Discussion | 23a | Provide a general interpretation of the results in the context of other evidence. | 4. Discussion |
|  | 23b | Discuss any limitations of the evidence included in the review. | 4.4 Strengths and Limitations |
|  | 23c | Discuss any limitations of the review processes used. | 4.4 Strengths and Limitations |
|  | 23d | Discuss implications of the results for practice, policy, and future research. | 4.4 Strengths and Limitations |
| **OTHER INFORMATION** | | |  |
| Registration and protocol | 24a | Provide registration information for the review, including register name and registration number, or state that the review was not registered. | PROSPERO CRD420261356354 |
|  | 24b | Indicate where the review protocol can be accessed, or state that a protocol was not prepared. | PROSPERO CRD420261356354 |
|  | 24c | Describe and explain any amendments to information provided at registration or in the protocol. | NA |
| Support | 25 | Describe sources of financial or non-financial support for the review, and the role of the funders or sponsors in the review. | Funding |
| Competing interests | 26 | Declare any competing interests of review authors. | Conflicts of Interest Statement |
| Availability of data, code and other materials | 27 | Report which of the following are publicly available and where they can be found: template data collection forms; data extracted from included studies; data used for all analyses; analytic code; any other materials used in the review. | Data Availability |

*From:*  Page MJ, McKenzie JE, Bossuyt PM, Boutron I, Hoffmann TC, Mulrow CD, et al. The PRISMA 2020 statement: an updated guideline for reporting systematic reviews. BMJ 2021;372:n71. doi: 10.1136/bmj.n71. This work is licensed under CC BY 4.0. To view a copy of this license, visit <https://creativecommons.org/licenses/by/4.0/>
